# Unveiling metabolic remodeling in mucopolysaccharidosis type III through integrative metabolomics and pathway analysis

**DOI:** 10.1186/s12967-018-1625-1

**Published:** 2018-09-04

**Authors:** Abdellah Tebani, Lenaig Abily-Donval, Isabelle Schmitz-Afonso, Bénédicte Héron, Monique Piraud, Jérôme Ausseil, Farid Zerimech, Bruno Gonzalez, Stéphane Marret, Carlos Afonso, Soumeya Bekri

**Affiliations:** 1 Department of Metabolic Biochemistry, Rouen University Hospital, 76000 Rouen Cedex, France; 2 Normandie Univ, UNIROUEN, CHU Rouen, INSERM U1245, 76000 Rouen, France; 3 Normandie Univ, UNIROUEN, INSA Rouen, CNRS, COBRA, 76000 Rouen, France; 4 Department of Neonatal Pediatrics, Intensive Care and Neuropediatrics, Rouen University Hospital, 76031 Rouen, France; 5 Department of Pediatric Neurology, Reference Center of Lysosomal Diseases, Trousseau Hospital, APHP and Sorbonne Université, GRC No 19, Pathologies Congénitales du Cervelet-LeucoDystrophies, AP-HP, Hôpital Armand Trousseau, 75012 Paris, France; 6 Service de Biochimie et Biologie Moléculaire Grand Est, Unité des Maladies Héréditaires du Métabolisme et Dépistage Néonatal, Centre de Biologie et de Pathologie Est, CHU de Lyon, Lyon, France; 7 INSERM U1088, Laboratoire de Biochimie Métabolique, Centre de Biologie Humaine, CHU Sud, 80054 Amiens Cedex, France; 8 Laboratoire de Biochimie et Biologie Moléculaire, Université de Lille et Pôle de Biologie Pathologie Génétique du CHRU de Lille, 59000 Lille, France

**Keywords:** Metabolomics, Inborn errors of metabolism, Mucopolysaccharidosis type III, Lysosomal storage diseases, Mass spectrometry, Ion mobility

## Abstract

**Background:**

Metabolomics represent a valuable tool to recover biological information using body fluids and may help to characterize pathophysiological mechanisms of the studied disease. This approach has not been widely used to explore inherited metabolic diseases. This study investigates mucopolysaccharidosis type III (MPS III). A thorough and holistic understanding of metabolic remodeling in MPS III may allow the development, improvement and personalization of patient care.

**Methods:**

We applied both targeted and untargeted metabolomics to urine samples obtained from a French cohort of 49 patients, consisting of 13 MPS IIIA, 16 MPS IIIB, 13 MPS IIIC, and 7 MPS IIID, along with 66 controls. The analytical strategy is based on ultra-high-performance liquid chromatography combined with ion mobility and high-resolution mass spectrometry. Twenty-four amino acids have been assessed using tandem mass spectrometry combined with liquid chromatography. Multivariate data modeling has been used for discriminant metabolite selection. Pathway analysis has been performed to retrieve metabolic pathways impairments.

**Results:**

Data analysis revealed distinct biochemical profiles. These metabolic patterns, particularly those related to the amino acid metabolisms, allowed the different studied groups to be distinguished. Pathway analysis unveiled major amino acid pathways impairments in MPS III mainly arginine–proline metabolism and urea cycle metabolism.

**Conclusion:**

This represents one of the first metabolomics-based investigations of MPS III. These results may shed light on MPS III pathophysiology and could help to set more targeted studies to infer the biomarkers of the affected pathways, which is crucial for rare conditions such as MPS III.

**Electronic supplementary material:**

The online version of this article (10.1186/s12967-018-1625-1) contains supplementary material, which is available to authorized users.

## Background

Metabolism is a complex, interconnected and finely regulated network. It is composed of reactions biochemical processes that transform endogenous or exogenous substrates into vital products for cell, tissue and organism function. As a result, deregulation of this homeostasis underlies the pathophysiological mechanisms of different diseases [1]. An alteration of a metabolic pathway may be related to nutritional, environmental, or genetic factors. Inborn errors of metabolism (IEM) are rare diseases mainly due to a genetic defect enzymes or cofactors involved in a metabolic pathway or in intra- or intercellular metabolites. For better management of IEM patients, rapid and accurate biochemical and molecular tests are needed. Omics are very appealing to speed up both their molecular understanding and may lead to more efficient biomarkers. Omics are very appealing to achieve holistic and systemic aspects of diseases [1]. The metabolome refers to all metabolites present in a given biological system [2]. Metabolomics is an “omics” technology that allows metabolome characterization [3, 4]. Metabolomics is particularly interesting in exploring IEM given their intrinsic with metabolism [5]. Lysosomal storage diseases (LSD) represent a group of about 50 inherited disorders related to deficient lysosomal proteins. This impairment leads to a progressive accumulation of metabolites or macromolecules within the lysosomales. This storage causes, at least partly, various organ failures [6]. Mucopolysaccharidoses (MPS) are a subgroup of LSD. They are related to impaired catabolism of glycosaminoglycans (GAGs), chondroitin sulfate (CS), dermatan sulfate (DS), heparan sulfate (HS), keratan sulfate (KS), and hyaluronan, leading to GAG accumulation in the lysosomes and extracellular matrix [7]. This accumulation leads to multiple progressive tissue and organ failures [8]. Seven distinct forms of MPS are described and related to 11 known enzyme deficiencies [6]. Overall incidence is more than 1 in 30,000 live births [9]. Most MPS patients are asymptomatic after birth, but prenatal symptoms may be observed in MPS I, MPS IVA, MPS VI and more frequently in MPS VII. Depending on the patient and the MPS subtype, symptoms and severity may vary. Different MPS treatments are either in clinical use or under clinical trials [10]. Mucopolysaccharidosis type III (MPS III), or Sanfilippo syndrome, is caused by a congenital deficiency of one of the four enzymes involved in the degradation of HS [11]. Four subtypes, MPS IIIA, MPS IIIB, MPS IIIC and MPS IIID, have an autosomal recessive inheritance [12]. Typically, patients with Sanfilippo disease present with no obvious clinical features prior to age 1–3 age years. Growth parameters may be higher compared to the reference range in the first years of life, while a growth delay may be observed in older patients. In all MPS III subtypes, central nervous system (CNS) involvement predominates (neurodegeneration, hyperactivity and behavioral disturbances) with less pronounced skeletal abnormalities and organomegaly. In addition, the clinical picture includes hirsutism, coarse facial features, cardiomegaly, thick hair, cloudy cornea, recurrent diarrhea, otitis and dysarthria [12–15]. MPS IIIA is the most severe type with an earlier onset and a rapid neurological deterioration. The first signs occur at around 1–3 years of age and the clinical symptoms worsen gradually and inevitably, resulting in the onset of severe dementia and a complete loss of motor functions. As other inherited metabolic diseases, the symptoms show high variability among patients even within the same family. Patients usually die before the third decade of life, although patients with a mild phenotype and allelic heterogeneity have been reported [12–15]. MPS IIIA (OMIM #252900) is caused by Heparane-*N*-sulfatase (SGSH, EC 3.10.1.1) deficiency with an incidence of 1 in 100,000 [16, 17]. MPS IIIB (OMIM #252920) is due to *N*-acetyl-α-glucosaminidase (NAGLU, EC 3.2.1.50) deficiency with an incidence of 1 in 200,000 [18]. MPS IIIC (OMIM #252930) is caused by heparan acetylCoA: α-glucosaminide-*N*-acetyltransferase (HGSNAT, EC 2.3.1.3) deficiency with an incidence of 1 in 1,500,000 [19]. MPS IIID (OMIM #252940) is due to *N*-acetylglucosamine-6-sulfatase (GNS, EC 3.1.6.14) deficiency with an incidence of 1 in 1,000,000 [20]. So far, no specific approved treatment is available. Gene therapy [21], bone marrow transplant [22], chaperon molecules [23], substrate deprivation therapy [24] and intrathecal enzyme therapy [25] are among the most active therapeutic research areas. The goal of this work is to apply both targeted and untargeted metabolomics on MPS IIIA, MPS IIIB, MPS IIIC and MPS IIID patients, compared to controls, to investigate metabolic changes in these conditions.

## Methods

### Urine samples

Random urine samples were collected from patients with a confirmed MPS diagnosis. Urine samples were collected within five expert centers for inherited metabolic diseases in France. The 49 untreated MPS III patients were evaluated as follows: 13 MPS IIIA patients: 6 males (age range from 5.1 to 12.0 years, mean age 6.2 years) and 7 females (age range from 1.9 to 18.4 years, mean age: 6.8 years); 16 MPS IIIB patients: 7 males (age range from 3.8 to 9.8 years, mean age 7.2 years) and 9 females (age range from 2.9 to 11.7 years, mean age 6.3 years); 13 MPS IIIC patients: 7 males (age range from 6.4 to 20.6 years, mean age:12.1 years) and 6 females (age range from 2.8 to 31.1 years, mean age 10.0 years); 7 MPS IIID patients: 3 males (age range from 3.8 to 17.5 years, mean age 8.9 years) and 4 females (age range from 3.4 to 18.7 years, mean age 7.8 years). Moreover, control urine samples were also collected from 66 healthy subjects, 27 males and 39 females (age range from 5.5 to 70 years, mean age 40.8 years). This project was approved by the Research Ethics Board of Rouen University Hospital (CERNI E2016-21).

### Metabolic phenotyping

The protocol used in this study has been previously described [26]. Briefly, urine samples were processed by transferring 200 μL of urine to 1.5 mL tubes and centrifuging at 4 °C for 10 min at 13,000*g*; then 100 µL of ultrapure water was added to 100 µL of supernatant and mixed. For untargeted metabolomics data acquisition, Ultraperformance liquid chromatography–ion mobility mass spectrometry on a Synapt G2 HDMS (Waters, Saint-Quentin-en-Yvelines, France) mass spectrometer as previously described [27]. Regarding targeted analysis, free amino acid profiles in urine was based on liquid chromatography coupled to a tandem mass spectrometry. Detailed protocols are presented in the Additional file 1.

### Data analysis

**Fig. 1:**
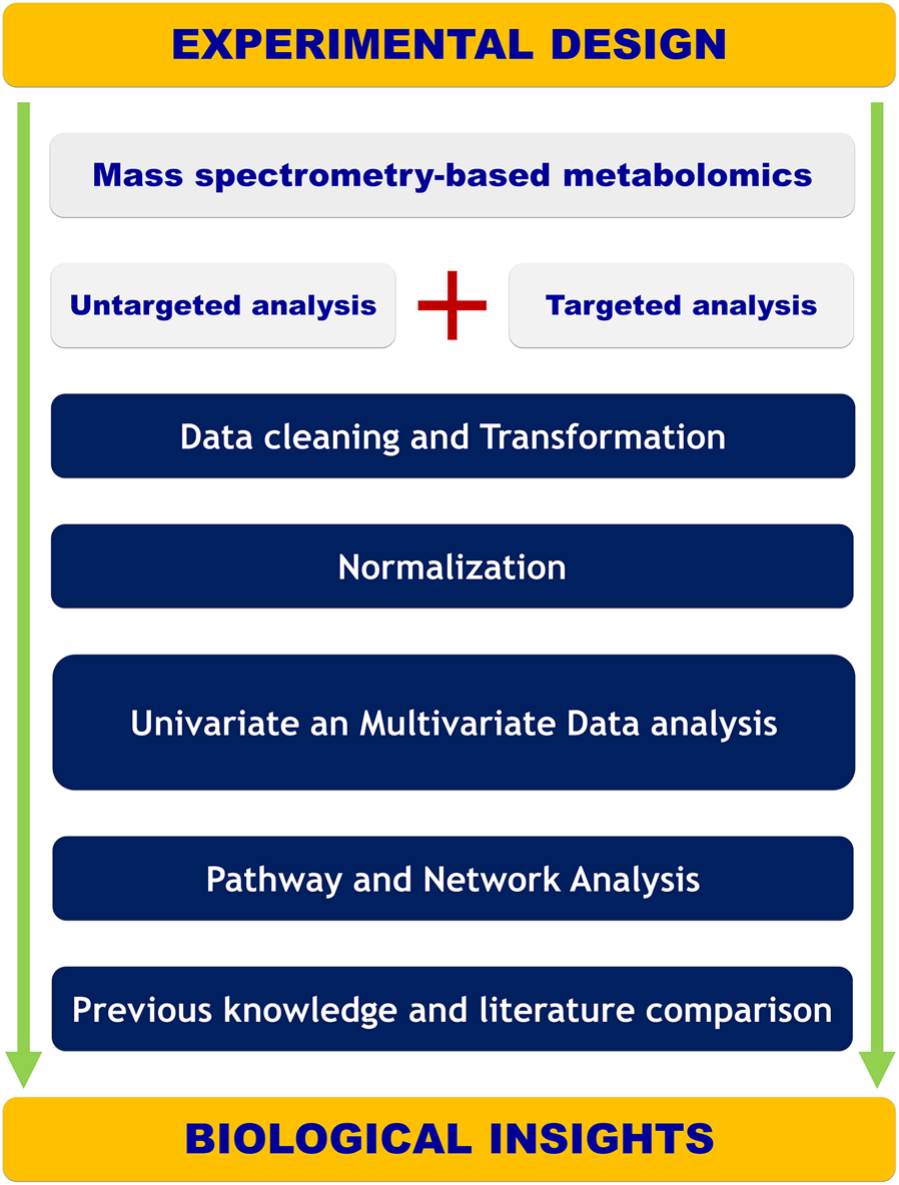
Illustration of the experimental workflow spanning from experimental design and data acquisition to pathway analysis and biological interpretation

A one-way analysis of variance (ANOVA) test was applied for multiple groups testing while a *t* test is used for binary comparisons. The Benjamini and Hochberg false discovery rate (FDR) method was used for multiple testing corrections with an FDR cut-off level of 5%. A Receiver operating characteristic curve (ROC) has been used to assess the diagnostic performance of the chosen classifiers. Support vector regression normalization for untargeted metabolomics data [28]. The normalized data has been log-transformed and pareto-scaled. All data modeling and analysis is done using SIMCA 15.0 (MKS DAS, Umeå, Sweden) and R software. The Mummichog algorithm has been used for pathway analysis [29] while MetaboAnalyst has been used for Metabolite Set Enrichment Analysis on the amino acid data [30]. Details regarding data modeling and validation results from all models are provided in Additional file 1. Figure 1 presents an overview of the implemented metabolomics workflow.

## Results

### Untargeted analysis

**Fig. 2:**
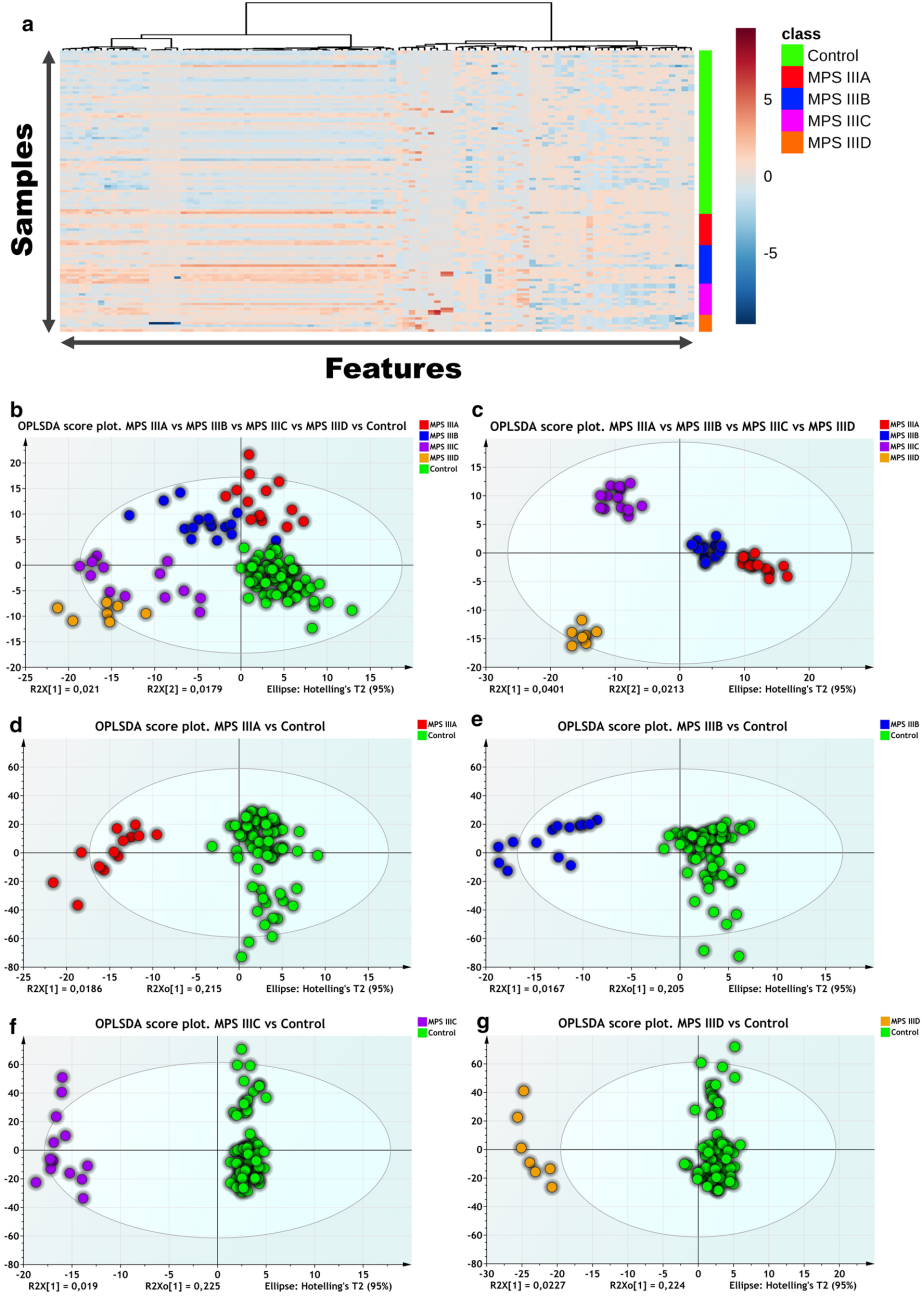
**a** Hierarchical cluster analysis and heat map visualization of top 100 variables (x-axis) ranked by ANOVA. The urine sample classes are represented along the y-axis. The color code was used to represent log-scaled intensities of features between − 5 (blue) and + 5 (brown), showing the relative abundance of the features according to the groups. **b** OPLSDA scores plot (R2 = 0.77, Q2 = 0.13) shows a clear separation between the different diseased and control groups (MPSIIIA, MPSIIIB, MPSIIIC and MPSIIID and control). **c** OPLSDA scores plot (R2 = 0.93, Q2 = 0.05) shows a clear separation between the different diseased groups (MPSIIIA, MPSIIIB, MPSIIIC and MPSIIIC). **d** Clear separation between MPSIIIA and control samples is observed (R2 = 0.89, Q2 = 0.23). **e** Clear separation of MPSIIIB samples from the controls is observed (R2 = 0.89, Q2 = 0.21). **f** Clear separation of MPSIIIC samples from the controls is observed (R2 = 0.98, Q2 = 0.39). **g** Clear separation of MPSIIID samples from the controls is observed (R2 = 0.95, Q2 = 0.36). Detailed model characteristics and validation are given in Additional file 1

The heatmap in Fig. 2a depicts the top 100 features ranked by ANOVA (*p* < 0.05 cut-off and FDR 5%). The results highlight correct clustering of the different sample groups and the dendrogram structure, using Euclidean distance, shows two main clusters of variables. We applied principal component analysis (PCA) to further analyze the underlying differential metabolic profiles. A three-component PCA model accounting for 18% of the total variance has been built. Trends, groups and potential outliers within the data are investigated using score plots. For predictive classification purposes, supervised methods are used since they allow the accurate modeling of the relationship between controls, MPS IIIA, IIIB, IIIC and IIID samples. First, an OPLS-DA classification was applied to the whole dataset. Samples were labeled according to the corresponding groups, MPS IIIA, IIIB, IIIC, IIID and control (Fig. 2b, c). A negative Q2 regression line intercept resulting from the permutation test allows the cross-validation of OPLSDA models. The final model had an R2 = 0.77 and Q2 = 0.13. The OPLS-DA scores plots (Fig. 2b) shows a clear separation, suggesting that the OPLS-DA model successfully classified samples according to their respective metabolic profiles. Model validation is assessed both by CV-ANOVA (*p*-value = 3 × 10^−2^) and by the permutation test (999 permutations gave a negative Q2 intercept). Details regarding model validation are shown in Additional file 1: Fig. S3). Furthermore, separate binary OPLS-DA classification models have been built for each disease sample vs control. For control and MPS IIIA samples the model had one predictive and two orthogonal components, and its validation parameters were as follows: R2 = 0.89, Q2 = 0.23 and CV-ANOVA *p*-value = 2.84 × 10^−3^ (Additional file 1: Fig. S4). The corresponding score plot is shown in Fig. 2d. It shows a clear separation between the two classes on the predictive component. For MPSIIIB and control samples the model had one predictive and two orthogonal components model with R2 = 0.89, Q2 = 0.21 and CV-ANOVA *p*-value = 5.28 × 10^−3^ (Fig. 2e). For MPSIIIC and control samples, the model has one predictive and three orthogonal components with R2 = 0.98, Q2 = 0.39 and CV-ANOVA *p*-value = 1.35 × 10^−5^ (Fig. 2f). Another OPLS-DA model was built for MPSIIID and control samples with one predictive and two orthogonal components model with R2 = 0.95, Q2 = 0.36 and CV-ANOVA *p*-value = 1.83 × 10^−5^ (Fig. 2g). To select discriminant variables, their respective VIP scores for each validated OPLS-DA model have been used. Based on 1 as a cutoff value, 25 features out of 854 were selected for the MPSIIIA VS Control model, 243 for MPSIIIB vs control, 247 for MPS IIIC vs control and 262 for the MPSIIID vs control model. The variables lists have been refined by retaining only the most discriminant variables and their putative annotation. The list included *N*-acetylserotonin, *N*-succinyl-l,l-2,6-diaminopimelate, octanoylglucuronide and **3**-2-hydroxyphenyl-propanoic acid. These discriminant variables are depicted in Tables 1 and 2 with their respective statistical metrics and annotation. Boxplots of the main discriminant features are presented in Additional file 1: Fig. S7. Using the area under the ROC curves (AUC), the discriminant performances of these features are also investigated. *N*-Acetylserotonin has the highest AUC for MPS IIIA (AUC = 0.83) and MPS IIIB (AUC = 0.83). *N*-Succinyl-l,l-2,6-diaminopimelate has the highest AUC (0.73) for MPS IIIC and octanoylglucuronide performed best for MPS IIID with an AUC = 0.79. The results are shown in Tables 1 and 2. Furthermore, the underlying impaired pathways in each disease are explored using Mummichog. The results are shown in Table 3. Interestingly, amino acid metabolisms and fatty acid pathways were markedly dysregulated.

**Table 1:** Some discriminant features, putatively annotated, extracted by the different OPLS-DA models for MPSIIIA, MPSIIIB, MPSIIIC and MPSIIID

HMDB	Putative annotation	Formula	*M*	m/z	Adduct	Δ m/z (ppm)	t_R_ (min)	tD (ms)	CCS (A^2^)	%RSD
HMDB01238	*N*-Acetylserotonin	C_12_H_14_N_2_O_2_	218.1055	241.0985	M + Na	13	5.44	2.65	146.0	16.76
HMDB12267	*N*-Succinyl-l,l-2,6-diaminopimelate	C_11_H_18_N_2_O_7_	290.1114	291.1223	M + H	10	7.48	3.24	162.5	9.90
HMDB33752	3-2-Hydroxyphenyl-propanoic acid	C_9_H_10_O_3_	166.0629	199.0970	M + CH_3_OH + H	3	6.77	2.38	138.8	21.89
HMDB10347	Octanoylglucuronide	C_14_H_24_O_8_	320.1471	303.1445	M − H_2_O + H	0	7.05	3.40	166.8	16.52

*M* monoisotopic mass, *ppm* parts per million, *t*_*R*_ retention time, *tD* drift time, *CCS* cross collision section, *VIP* variable importance in projection

**Table 2:** Statistical and discriminant metrics of the selected annotated features

HMDB	Putative annotation	MPSIIIA			MPSIIIB			MPSIIIC			MPSIIID		
		FDR	AUC	VIP	FDR	AUC	VIP	FDR	AUC	VIP	FDR	AUC	VIP
HMDB01238	*N*-Acetylserotonin	*2.28E−03*	*0.83*	*1.21*	*6.05E−01*	*0.83*	*0.93*	8.61E−01	0.61	0.99	9.70E−01	0.54	0.35
HMDB12267	*N*-Succinyl-l,l-2,6-diaminopimelate	3.88E−01	0.60	0.56	7.44E−01	0.63	0.64	*9.06E−03*	*0.73*	*1.29*	6.56E−01	0.69	0.05
HMDB33752	3-2-Hydroxyphenyl-propanoic acid	*1.76E−02*	*0.76*	*1.53*	5.30E−01	0.77	1.76	3.31E−01	0.66	0.93	7.56E−01	0.62	0.22
HMDB10347	Octanoylglucuronide	6.75E−01	0.58	0.69	3.86E−01	0.72	1.95	8.54E−01	0.64	0.51	*2.57E−02*	*0.79*	*1.90*

*FDR* false discovery rate, *AUC* area under the curve, *VIP* variable importance in projection

Significant features are highlighted in italics for control and each disease comparison (false discovery rate FDR = 5%)

**Table 3:** Significantly dysregulated pathways

	Pathway	Overlap size	*p*-value (FDR = 5%)
MPS IIIA	Vitamin B1 (thiamin) metabolism	2	2.77E−03
	Pyrimidine metabolism	2	9.86E−03
MPS IIIB	TCA cycle	2	1.41E−03
	Aspartate and asparagine metabolism	4	1.81E−03
	Vitamin E metabolism	3	3.00E−03
	Methionine and cysteine metabolism	3	4.78E−03
	Fatty acid activation	2	4.89E−03
	Lysine metabolism	2	9.66E−03
	De novo fatty acid biosynthesis	2	1.31E−02
	Tryptophan metabolism	3	3.31E−02
MPS IIIC	Vitamin B1 (thiamin) metabolism	2	5.54E−04
	Omega-3 fatty acid metabolism	2	8.27E−04
	Butanoate metabolism	2	1.71E−03
	Tryptophan metabolism	3	8.07E−03
	Linoleate metabolism	2	1.18E−02
	Tyrosine metabolism	3	2.28E−02
	Methionine and cysteine metabolism	2	2.60E−02
MPS IIID	TCA cycle	2	1.41E−03
	Vitamin B1 (thiamin) metabolism	2	1.41E−03
	Aspartate and asparagine metabolism	4	3.08E−03
	Butanoate metabolism	2	5.14E−03
	Carnitine shuttle	2	2.32E−02
	Arginine–proline metabolism	2	2.91E−02
	Tryptophan metabolism	3	4.36E−02

*FDR* false discovery rate

### Targeted analysis

**Fig. 3:**
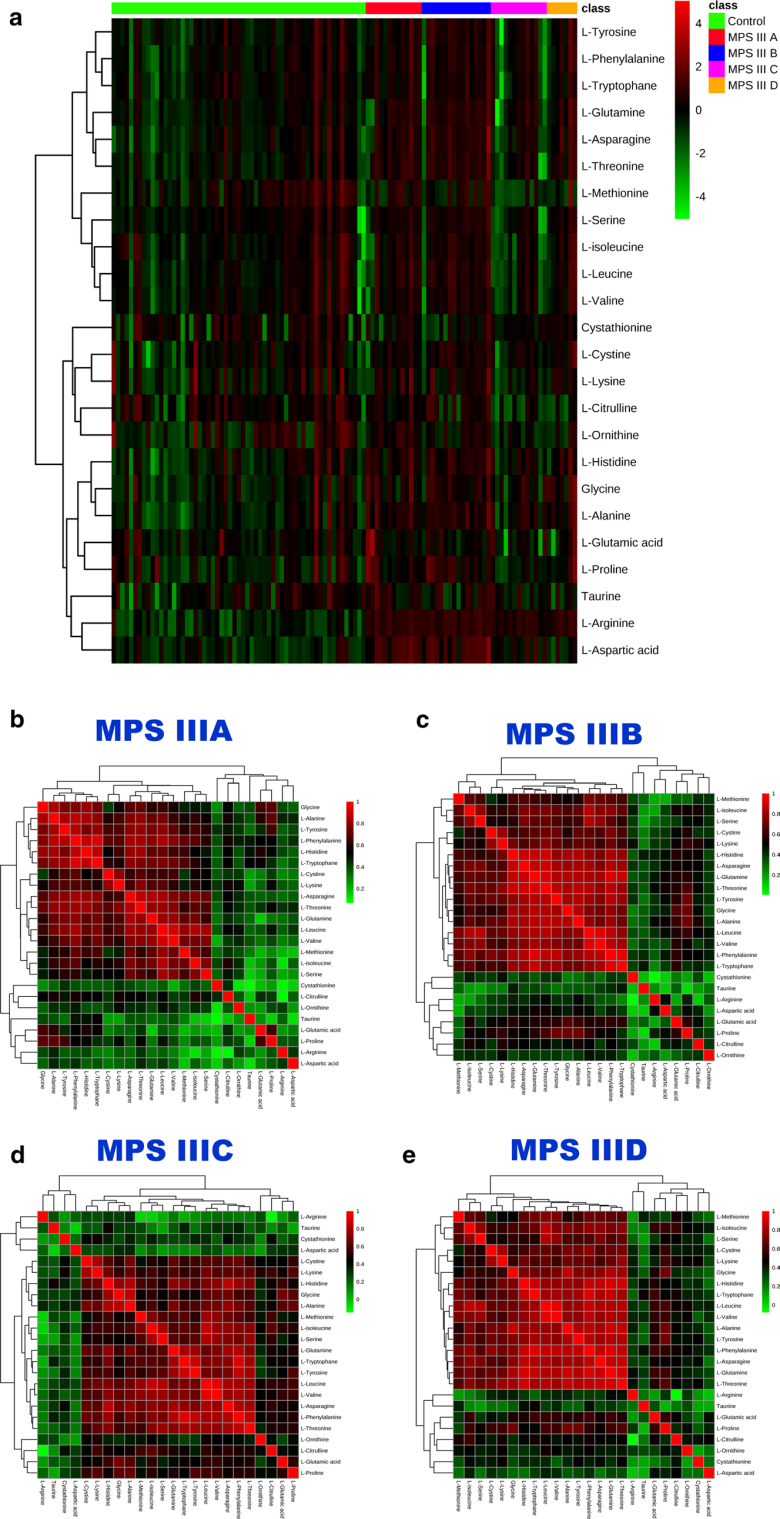
**a** Heat map representing the clustering of 24 amino acids across the five groups of samples (MPS IIIA, MPS IIIB, MPS IIIC, MPS IIID and Controls). Columns represent individual samples and rows refer to amino acid. Shades of green or red represent elevation or decrease, respectively, of an amino acid. **b**–**e** Spearman rank-order correlation matrix 24 amino acids based on their concentrations profiles across all samples in MPS IIIA, MPS IIIB, MPS IIIC and MPS IIID respectively. Shades of green to red represent low-to-high correlation coefficient between markers

**Fig. 4:**
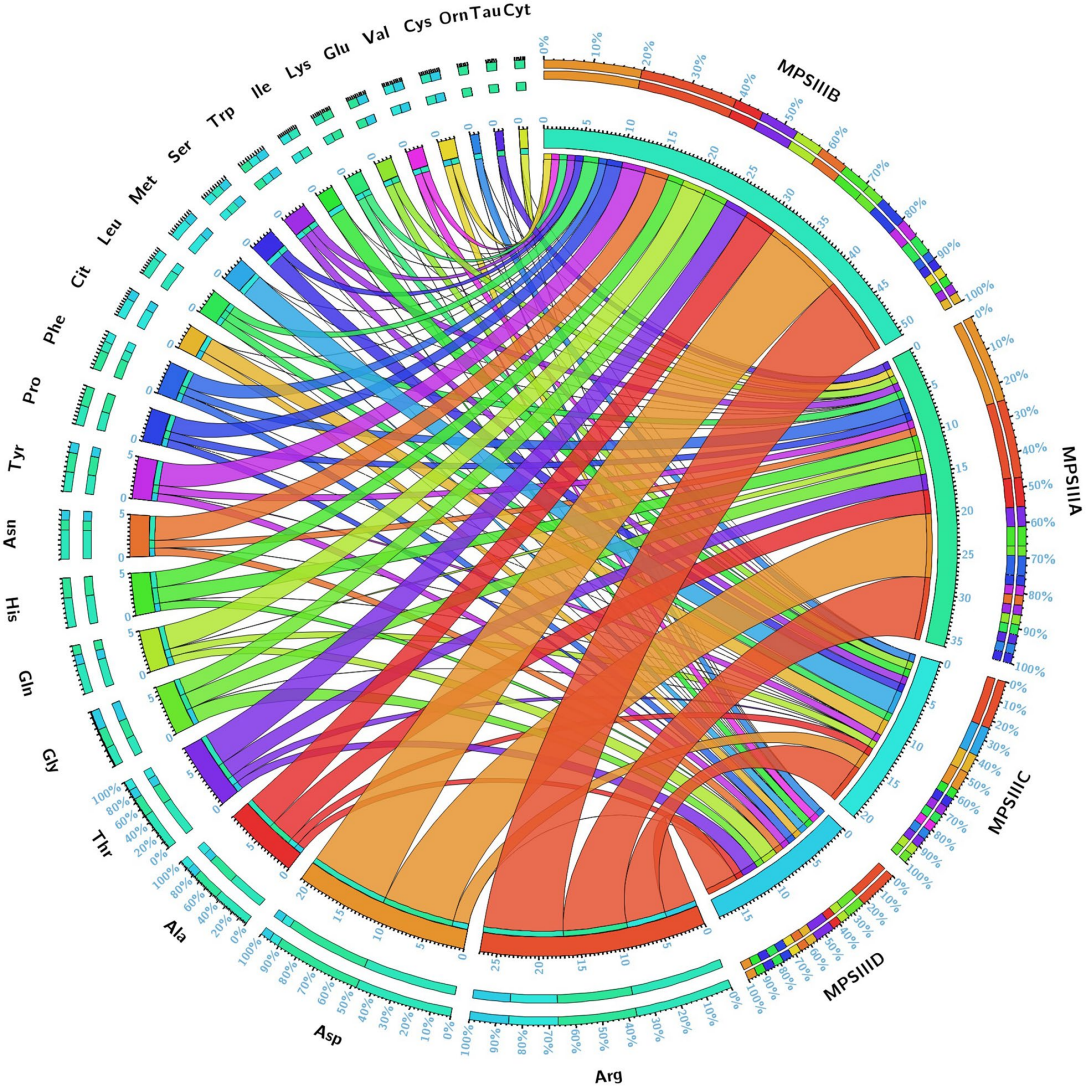
Circular plot of the 24 amino acids and their related −log (*p*) values in the different studies MPS III groups. Segments are color-coded according to amino acids and ribbon size represents −log (*p*) values (large ribbons mean low *p*-values). Corresponding *p*-values are presented in Table 4

**Fig. 5:**
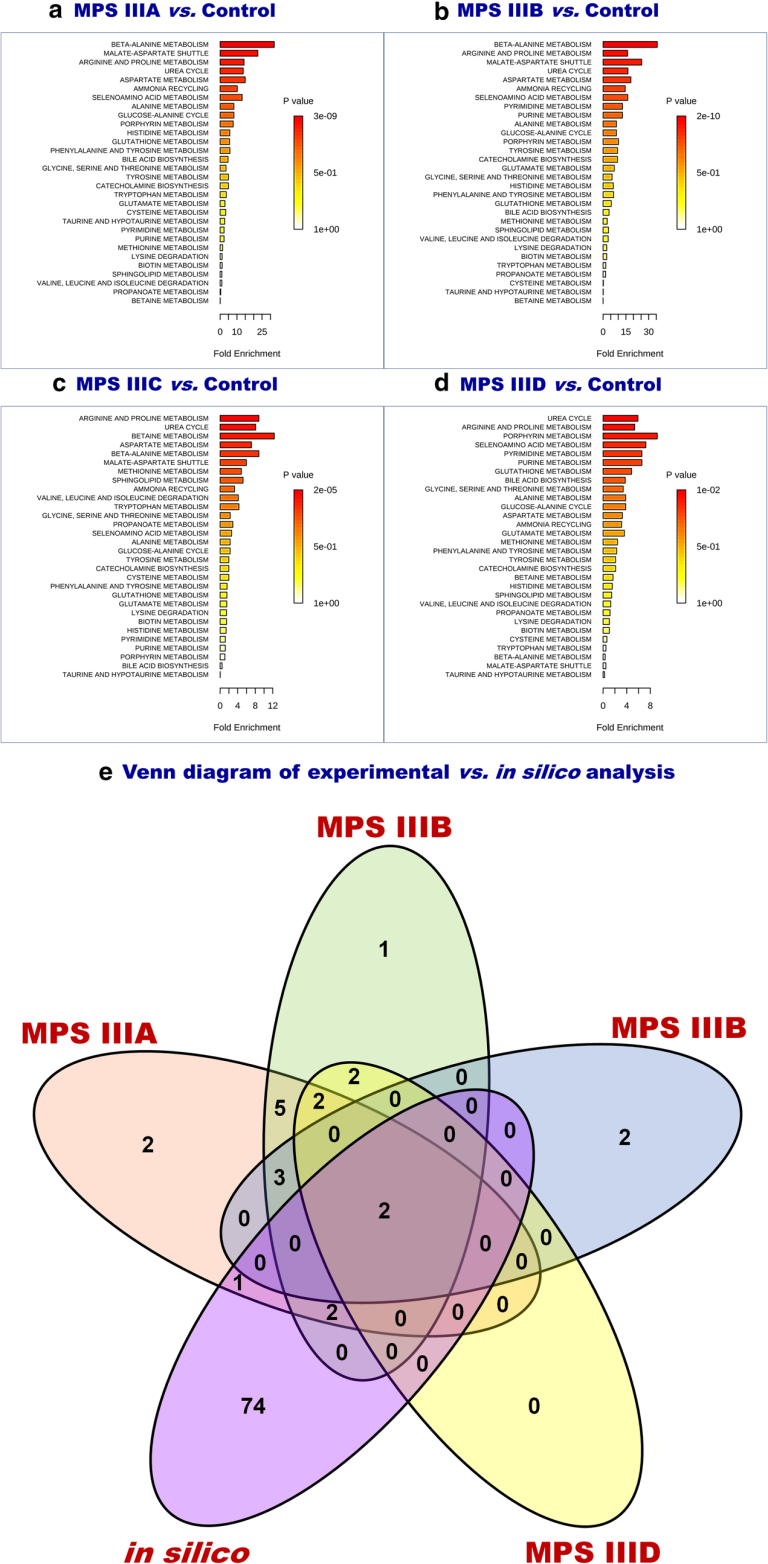
Metabolite Set Enrichment Analysis using amino acid concentrations. **a** MPS IIIA vs Control. **b** MPS IIIB vs Control. **c** MPS IIIC vs Control. **d** MPS IIID vs Control. **e** Venn diagram of the significant pathways retrieved from experimental metabolomics data and in silico systems biology approach from Salazar et al. [37]. The diagram shows two common metabolisms: arginine–proline metabolism and urea cycle. Detailed pathway information is given in Additional file 1: Table S6

We also quantified twenty-four amino acids and Additional file 1: Table S4 presents their absolute urine concentrations. Boxplots of normalized amino acid concentrations are shown in Additional file 1: Fig. S8 and the statistical metrics are presented in Table 4. MPS IIIA yielded 11 significantly changed amino acids compared to controls: arginine, aspartic acid, alanine, threonine, histidine, phenylalanine, glycine, proline, asparagine and tyrosine. For MPS IIIB vs control, arginine, aspartic acid, alanine, threonine, histidine, phenylalanine, glycine, proline, glutamine, asparagine, tyrosine and leucine showed significant differences. regarding MPS IIIC vs control, 6 amino acids showed differences: arginine, aspartic acid, serine, isoleucine, methionine and citrulline. For MPS IIID vs control, 6 amino acids showed differences: arginine, alanine, threonine, glycine, glutamine and citrulline. To holistically determine the amino acid profile differences between controls and each of the MPS III subtype patients, the amino acids concentrations were assessed using an ANOVA test. The analysis yielded 17 amino acids above the *p* < 0.05 cut-off (FDR 5%). A hierarchical clustering analysis was applied to group samples according to their profile similarities. The heatmap in Fig. 3a represents the 24 amino acids ranked by ANOVA. The results show that all samples belonging to the same group were correctly clustered. The dendrogram structure, using Euclidean distance, highlights two main clusters of variables. Furthermore, a correlation analysis has been performed. Figure 3b–e presents the heatmap of the correlation analysis for MPS IIIA, MPS IIIB, MPS IIIC and MPS IIID, respectively. Both figures show a clear cluster of variables that have high correlation. Figure 3b (MPS IIIA vs control) shows a main cluster including alanine, leucine, valine, glycine, tyrosine, threonine, isoleucine, histidine, lysine, tryptophan, serine, asparagine, glutamine, phenylalanine, cystine and methionine. Regarding MPS IIIB vs control, Fig. 3c shows two main clusters: the main one includes methionine, isoleucine, serine, cystine, lysine, histidine, asparagine, glutamine, threonine, tyrosine, glycine, alanine, leucine, valine, phenylalanine, and tryptophan. Regarding MPS IIIC vs control, Fig. 3d shows three clusters: the main one includes cystine, lysine, histidine, glycine, alanine, methionine, isoleucine, serine, glutamine, tryptophan, tyrosine, leucine, valine, asparagine, phenylalanine and threonine. For MPS IIID vs control, Fig. 3e shows two clusters: the main one includes methionine, isoleucine, serine, cystine, lysine, glycine, histidine, tryptophan, leucine, valine, leucine, valine, tyrosine, phenylalanine, asparagine, glutamine and threonine. To assess the diagnostic performance of the different amino acids, we performed univariate ROC curve analyses for the different MPS III subtype compared to controls. For MPS IIIA, there were four amino acids with a high AUC above 0.80, including: arginine (0.98), aspartic acid (0.95), alanine (0.85) and threonine (0.81). The same procedure was performed for MPS IIIB vs control and indicated seven amino acids with a high AUC above 0.80 and these were: arginine (0.98), aspartic acid (0.94), Alanine (0.87) and threonine (0.86), histidine (0.81), glutamine (0.87), asparagine (0.83). For MPS IIIC, the results showed only Arginine with a high AUC (0.95). Regarding MPS IIID, three amino acids showed a high AUC: arginine (0.98), alanine (0.81) and glycine (0.81). The overall univariate and ROC analysis results are shown in Table 4 and Fig. 4. The ROC curves along with a comparison of the different combinations of the main significant amino acids have been performed using PLSDA models with three components each. The results are presented in Additional file 1: Fig. S9. Pathway analysis yielded the main impaired metabolisms. For MPS IIIA vs control and MPS IIIB vs control analyses, beta-alanine metabolism, malate–aspartate shuttle, arginine–proline, urea cycle and aspartate metabolism were among the most affected pathways. For the MPS IIIC vs control analysis, methionine metabolism, in addition to the abovementioned metabolic pathways, was the most affected. For the MPS IIID vs control analysis, urea cycle, arginine–proline metabolism, porphyrin metabolism and pyrimidine and purine metabolism were the most affected pathways. The overall results are shown in Fig. 5a–d for all the studied groups.

**Table 4:** Fold change, *t*-test statistics, and area under the curve (AUC) of the receiver operating curves (ROC) for 24 amino acids, free carnitine and acylcarnitines (*p *< 0.05)

	MPSIIIA vs control			MPSIIIB vs control			MPSIIIC vs control			MPSIIID vs control		
	AUC	q-value (FDR)	Fold change	AUC	q-value (FDR)	Fold change	AUC	q-value (FDR)	Fold change	AUC	q-value (FDR)	Fold change
l *-Arginine*	*0.98*	*7.77E−09*	*− 4.53*	*0.98*	*1.45E−10*	*− 3.90*	*0.95*	*4.26E−06*	*− 3.60*	*0.98*	*1.08E−04*	*− 3.75*
l *-Aspartic acid*	*0.95*	*2.10E−09*	*− 3.83*	*0.94*	*1.29E−10*	*− 3.30*	*0.77*	*1.43E−02*	*− 1.45*	0.56	6.06E−01	0.30
l *-Alanine*	*0.86*	*1.32E−03*	*− 1.11*	*0.87*	*1.81E−04*	*− 0.45*	0.67	2.55E−01	− 0.33	*0.81*	*4.63E−02*	*− 0.50*
l *-Threonine*	*0.81*	*8.18E−03*	*− 1.13*	*0.86*	*2.68E−04*	*− 0.72*	0.50	2.86E−01	0.91	*0.78*	*4.63E−02*	*− 0.61*
l *-Histidine*	*0.77*	*1.76E−02*	*− 0.98*	*0.81*	*1.60E−03*	*− 0.43*	0.59	5.02E−01	− 0.11	0.69	2.89E−01	− 0.09
l *-Phenylalanine*	*0.76*	*2.82E−02*	*− 0.78*	*0.77*	*6.94E−02*	*0.08*	0.52	4.02E−01	0.67	0.63	2.89E−01	− 0.03
*Glycine*	*0.75*	*1.76E−02*	*− 0.82*	*0.78*	*3.34E−03*	*− 0.13*	0.63	4.09E−01	− 0.12	*0.81*	*2.27E−02*	*− 0.69*
l *-Proline*	*0.73*	*5.17E−02*	*− 0.69*	*0.77*	*3.93E−03*	*− 0.16*	0.52	6.61E−01	0.44	0.71	2.89E−01	0.03
l *-Glutamine*	0.73	1.98E−01	− 0.46	*0.87*	*1.04E−03*	*− 0.49*	0.55	4.02E−01	0.77	*0.72*	*4.63E−02*	*− 0.52*
l *-Asparagine*	*0.72*	*7.76E−02*	*− 0.69*	*0.83*	*1.48E−03*	*− 0.51*	0.55	6.61E−01	0.46	0.70	1.67E−01	− 0.30
l *-Tyrosine*	*0.71*	*6.88E−02*	*− 0.63*	*0.80*	*4.01E−03*	*− 0.21*	0.50	2.86E−01	0.89	0.64	2.97E−01	0.04
l-Tryptophan	0.70	1.19E−01	− 0.49	0.74	2.59E−01	0.36	0.58	1.29E−01	1.13	0.55	5.60E−01	0.44
l *-Leucine*	0.67	1.84E−01	− 0.49	*0.79*	*2.31E−02*	*− 0.08*	0.64	2.55E−01	0.93	0.62	3.62E−01	0.08
Taurine	0.66	1.84E−01	− 0.71	0.52	8.38E−01	0.73	0.56	9.70E−01	0.27	0.55	6.53E−01	1.12
l *-Serine*	0.65	4.20E−01	− 0.44	0.78	1.01E−01	− 0.20	*0.63*	*8.53E−02*	*1.89*	0.68	3.73E−01	− 0.22
l-Glutamic acid	0.62	1.34E−01	− 0.48	0.55	8.23E−01	0.78	0.52	2.86E−01	0.76	0.52	5.37E−01	1.12
l-Lysine	0.61	3.99E−01	− 0.43	0.65	2.01E−01	0.10	0.59	3.72E−01	0.88	0.66	4.09E−01	0.02
l *-Isoleucine*	0.61	7.85E−01	− 0.02	0.67	2.51E−01	0.36	*0.68*	*4.42E−02*	*1.35*	0.59	4.09E−01	0.18
l-Valine	0.60	6.68E−01	− 0.10	0.70	2.64E−01	0.36	0.66	2.55E−01	0.99	0.58	4.09E−01	0.15
l-Ornithine	0.60	2.65E−01	− 1.01	0.53	8.53E−01	0.70	0.61	2.86E−01	1.53	0.53	6.53E−01	1.31
l-Cystine	0.59	6.68E−01	− 0.19	0.71	1.01E−01	− 0.08	0.51	9.70E−01	0.32	0.69	2.89E−01	− 0.38
Cystathionine	0.55	9.48E−01	0.25	0.60	5.76E−01	1.36	0.63	9.70E−01	0.25	0.53	5.60E−01	0.09
l *-Methionine*	0.54	9.48E−01	0.11	0.51	9.71E−01	0.83	*0.79*	*3.29E−03*	*2.15*	0.69	3.62E−01	1.60
l *-Citrulline*	0.52	8.20E−01	0.03	0.60	6.61E−01	0.54	*0.74*	*9.43E−03*	*2.11*	*0.76*	*7.15E−02*	*2.48*

Significant features are highlighted in italics (false discovery rate FDR = 5%)

## Discussion

In this study, MPS III urine patterns of metabolites have been studied to unveil the biochemical indicators that may differentiate MPS III patients from control individuals. Of note, the mean-age difference between the studied groups represent a drawback which is mainly due to pediatric recruitment difficulties and ethical considerations. However, given the stringency of the statistical cut-off and the applied multiple testing correction might circumvent some of these biases. Using untargeted metabolomics, we succeeded in building a predictive model with a clear separation between the different studied groups—MPS IIIA, MPS IIIB, MPS IIIC, MPS IIID and control sample—which is underlined by the metabolic pattern similarity in each group. The retrieved data revealed a profound metabolic modeling mainly of amino acid-related metabolism. In light of these results, targeted amino acid analysis has been performed, which confirmed the deep metabolic alterations. Using these data, pathway analysis succeeded in identifying the main disrupted pathways. Salazar et al. [31] reported a genome-scale human metabolic reconstruction based approach to understand the effect of metabolism alterations in MPS. This in silico approach applied to MPS III subtypes (MPS IIIA, MPS IIIB, MPS IIIC and MPS IIID) by silencing, respectively, *SGSH, NAGLU, HGSNAT* and *GNS* genes, allowed the generation of models which were analyzed through flux balance and variability analysis. We performed a comparative analysis between the in silico systems based analysis data and the pathway analysis results of this present study. This comparison is illustrated by a Venn diagram (Fig. 5e) and showed two main common metabolisms: arginine–proline metabolism and urea cycle. Detailed data are presented in Additional file 1: Table S4. Arginine–proline metabolism and it connections to urea cycle is depicted in Additional file 1: Fig. S10.

As observed in MPS I patients [26] the arginine metabolism is the most altered pathway, aspartic acid is highly elevated in MPS IIIA and IIIB, significantly elevated in IIIC and shows a rising tendency in IIID. These metabolisms (arginine–proline, urea cycle, aspartic acid) have been reported to be upregulated along with high autophagic activity upon oxygen and glucose reduction using cultured fibroblasts [32] which is consistent with their involvement in bioenergetic balance. As in other LSDs, arginine metabolism may be challenged in MPS III due to lysosome dysfunction and its subsequent autophagic block [33]. Aspartic-acid contributes to the synthesis of *N*-acetyl-l-aspartate (NAA) and its derivative *N*-acetylaspartylglutamate (NAAG). NAA plays a central role in neuronal osmosis and myelin synthesis whereas NAAG is a key neurotransmitter. NAA and NAAG are highly present in the brain; their synthesis and catabolism take place in the brain and are highly regulated and compartmentalized [34]. This high-level homeostasis is consistent with a key function of these components in the central nervous system. Thus, the impact of NAA metabolism is illustrated by the brain damages associated with the NAA catabolic enzyme called aspartoacylase in Canavan disease, an early-onset spongiform leukodystrophy [35]. It has also been reported that the NAA signal obtained using magnetic resonance spectroscopy is reduced in metachromatic leukodystrophy, Krabbe disease and other lysosomal storage diseases [36, 37].

A recent study reported metabolomics profiling in serum from MPS IIIA and MPS IIIB patients. Our results are in accordance with this study, which showed notable metabolic disturbance of key amino acids indicating profound metabolic pathway remodeling. Interestingly, NAA levels were decreased in these patients compared to the control patients [38].

## Conclusion

In this study, urine global metabolomics profiling revealed profound metabolic impairments in patients with MPS III. The identification of pathological metabolomics signatures may provide better understanding of the pathophysiological mechanism underlying these diseases and thus allow therapeutic innovation in such rare conditions.

## Additional file

**Additional file 1.** Detailed analytical protocols and data modeling.

## Abbreviations

IEM: inborn errors of metabolism
LSD: lysosomal storage diseases
MPS: mucopolysaccharidoses
GAGs: glycosaminoglycans
MPS III: mucopolysaccharidosis type III
UPLC–IM-MS: ultraperformance liquid chromatography–ion mobility mass spectrometry
CCS: collision cross section
ERT: enzyme replacement therapy
QC: quality control
HS: heparan sulfate
DS: dermatan sulfate
KS: keratan sulfate
ROC: receiver operating characteristic
FDR: false discovery rate
PCA: principal component analysis
OPLS-DA: orthogonal partial least-squares-discriminant analysis
VIP: variable influence in projection
AUC: area under curve
