# Supplementary material for: Unveiling metabolic remodeling in mucopolysaccharidosis type III through integrative metabolomics and pathway analysis
Source: J Transl Med. 2018 Sep 4;16:248. doi: 10.1186/s12967-018-1625-1 (PMC6122730; doi:10.1186/s12967-018-1625-1)
Supplement: Supplementary file 1 — Additional file 1. Detailed analytical protocols and data modeling. [file 12967_2018_1625_MOESM1_ESM.pdf]

Supplementary material for:

Unveiling metabolic remodeling in mucopolysaccharidosis type III through integrative metabolomics and pathway analysis

Abdellah Tebani[1,2,3], Lenaig Abily-Donval[2,4], Isabelle Schmitz-Afonso[3], Bénédicte Héron[5], Monique Piraud[6], Jérôme Ausseil[7], Farid Zerimech[8], Stéphane Marret[2,4], Carlos Afonso[3], Soumeya Bekri[1, 2,*]

[1] Department of Metabolic Biochemistry, Rouen University Hospital, Rouen, 76000, France
[2] Normandie Univ, UNIROUEN, CHU Rouen, INSERM U1245, 76000 Rouen, France
[3] Normandie Univ, UNIROUEN, INSA Rouen, CNRS, COBRA, 76000 Rouen, France
[4] Department of Neonatal Pediatrics and Intensive Care, Rouen University Hospital, Rouen, 76031, France
[5] Department of Pediatric Neurology, Reference Center of Lysosomal Diseases, Trousseau Hospital, APHP, and GRC ConCer-LD, Sorbonne Universities, UPMC University 06, Paris, France
[6] Service de Biochimie et Biologie Moléculaire Grand Est, Unité des Maladies Héréditaires du Métabolisme et Dépistage Néonatal, Centre de Biologie et de Pathologie Est CHU de Lyon, Lyon, France.
[7] INSERM U1088, Laboratoire de Biochimie Métabolique, Centre de Biologie Humaine, CHU Sud, 80054, Amiens Cedex, France.
[8] Laboratoire de Biochimie et Biologie Moléculaire, Université de Lille et Pôle de Biologie Pathologie Génétique du CHRU de Lille, 59000, Lille, France.

Corresponding author:
Prof. Soumeya BEKRI
Department of Metabolic Biochemistry,
Rouen University Hospital 76031 ROUEN Cedex France
soumeya.bekri@chu-rouen.fr
Tel 00 33 2 32 88 81 24
Fax 00 33 2 32 88 83 41

Contents

Technical details

1. **Reagents**
2. **LC-ESI-MS/MS methodology for determination of amino acids**
3. **UHPLC-IM-MS analysis**
   - **3.1. Data acquisition**
   - **3.2. Quality Control**
   - **3.3. Data analysis and modelling**
   - **3.4. Feature selection and annotation**
   - **3.5. Pathway analysis**

Tables

Table S1. MRM transitions for each amino acid and its corresponding internal standard.
Table S2. Instrumental settings for UHPLC-IM-MS analysis.
Table S3. The normalized concentrations of free amino acids in urine samples of the studied groups.
Table S4. Data for Venn diagram of the significant pathways retrieved from untargeted, targeted approaches and *in silico* systems biology.

Figures

Figure S1. Illustration of the analysis sequence.
Figure S2. OPLSDA model validation including the three groups: MPS I, MPSIT and Controls.
Below the figure, model parameters and CV-ANOVA results are presented.
Figure S3. OPLSDA model validation for MPS IIIA vs Control
Figure S4. OPLSDA model validation for MPS IIIB vs Control
Figure S5. OPLSDA model validation for MPS IIIC vs Control
Figure S6. OPLSDA model validation for MPS IIID vs Control
Figure S7. Boxplots of some selected discriminant features in the different groups
Figure S8. Boxplots of amino acid concentrations across the five studied groups: MPS IIIA, MPS IIIB, MPS IIIC, MPS IIID and Control samples.
Figure S9. Area under the receiver operating characteristic (ROC) curves, comparing diagnostic performance of the most significant quantified Arginine to differentiate the different MPS III subtypes and Control samples.
Figure S10. Pathways of arginine metabolism and its connections to urea cycle.

1. Reagents and chemicals

Acetonitrile was purchased from VWR Chemicals (France), ultrapure water (18 MX) from Millipore (Molsheim, France) and formic acid from Fluka (Saint Quentin Fallavier, France). The chemicals used were of analytical grade. Leucine Enkephalin (Sigma–Aldrich) at a concentration of 2 ng/µL (in acetonitrile/water, 50/50) was used as reference for mass measurements. Poly-DL-alanine was prepared in 50:50 (v/v) water/acetonitrile at 10 mg/L and used for ion mobility cell calibration. The aTRAQ Kit for Amino acid Analysis of Physiological Fluids (Ref. 4442674) was purchased from Sciex (Life Science Holdings, France). MassChrom® Amino Acids and Acylcarnitines kit (Ref. 55000) was purchased from Chromsystems (Gräfelfing Germany). HPLC gradient grade methanol was purchased from VWR Chemicals (Fontenay-sous-Bois, France).

2. LC-ESI-MS/MS methodology for determination of amino acids

The aTRAQ Kit for Amino acid Analysis of Physiological Fluids was purchased from Sciex (Framingham, MA, USA). It consisted of amine-modifying labeling aTRAQ reagent Δ8, aTRAQ internal standard set of amino acids labeled with the aTRAQ reagent Δ0, 10 % sulfosalicylic acid, borate buffer of pH 8.5, 1.2 % hydroxylamine and mobile phase modifiers – formic acid and heptafluorobutyric acid. HPLC gradient grade methanol was purchased from J.T. Baker (Center Valley, PA, USA). Deionized water obtained from Millipore Simplicity UV water purification system (Waters Corporation, Milford, MA, USA) was used. Amino acids standards were purchased from SIGMA-ALDRICH (acidic and neutral amino acids Ref: A6407-5ML. Basic amino acids Ref: A6282-5ML).
The following protocol was used for preparation of urine samples. The urine were first diluted using 50/50 v/v using deionized water. An aliquot of 40 µL of the sample was added to 10 µL of 10% sulfosalicylic acid in order to precipitate proteins. After mixing and centrifugation (10 000 x g for 2 min) the supernatant was mixed with 40 µL of borate buffer. Next, an aliquot of 10 µL the obtained solution was labeled with aTRAQ reagent solution (aTRAQ reagent Δ8), mixed and centrifuged. After 30 min of incubation at room temperature the labeling reaction was stopped by addition of 5 µL 1.2% hydroxylamine solution and the sample was incubated at room temperature for 15 min. In the next step, 32 µL of the internal standard solution was added to the sample. After mixing and centrifugation the sample was evaporated in a vacuum concentrator for 15 min in order to reduce volume to about 20 µL. Then the residue was diluted with 20 µL of water. Each determined amino acid had its corresponding internal standard (the same amino acid labeled with the aTRAQ reagent Δ0). Two non-proteinogenic amino acids (norleucine and norvaline) were used to evaluate the labeling efficiency and recovery. A calibration curve was constructed using five concentrations derived from the peak area ratio of each amino acid and the internal standard. A calibration curve has been used as follows:

**Amino acids (µM)**	5	25	100	250	500	1200
**Glutamine (µM)**	10	50	200	500	1000	2500
**Cystine (µM)**	5	25	100	250	500	

Concentrations were calculated from these area ratios using the calibration curve established by simple regression. The determination of free amino acid levels was conducted using the liquid chromatography instrument Prominence Shimadzu UFLC system consisting of a DGU-20A3 degasser, a LC-20AB pump, a SIL-20ACHT autosampler, and a CTO-20AC oven (Shimadzu, Prominence, Kyoto, Japan) coupled to the 4000 QTRAP mass spectrometer (Sciex, Framingham, MA, USA) with an electrospray ion source. The chromatographic separation was achieved on Sciex C18 column (5 µm, 4.6 mm x 150 mm) maintained at 50 °C with a flow rate of 800 µL/min. A mobile phase gradient of eluent A (0.1% formic acid and 0.01% heptafluorobutyric acid in water) and eluent B (0.1% formic acid and 0.01% heptafluorobutyric acid in methanol) was applied. A gradient profile was the following: from 2% to 40% of B from 0 till 6 min, maintained at 40% of B for 4 min, then increased to 90% of B till 11 min and held at 90% of B for 1 min. After 12 min the gradient decreased to 2% of B. From 13 to 18 min, the mobile phase composition was unaltered. The injection volume was set at 2 µL. The ion source settings were: curtain gas, 20 psig; ion spray voltage, 4500 V; source temperature, 600 °C; ion source gas 1, 60 psig and ion source gas 2, 50 psig. The mass spectrometer operated in positive ionization mode with the following parameters: entrance potential, 10 V; declustering potential, 30 V and collision cell exit potential, 5 V. Collision energy of 30 eV was applied. The list of measured MRM transitions is presented in **Table S1**. Scheduled multiple reaction monitoring mode was used with nitrogen as a collision gas. A system suitability test was conducted before each batch of the samples (analysis of a standard mixture) to warm up the LC-MS/MS system and check the inter-day performance of the system. Data acquisition and processing were performed using the Analyst 1.5 software (Sciex, Framingham, MA, USA).

Table S1. MRM transitions for each amino acid and its corresponding internal standard.

	Analyte		Internal standard	
**Amino acid**	**Q1 (*m/z*)**	**Q2 (*m/z*)**	**Q1 (*m/z*)**	**Q2 (*m/z*)**
**Alanine**	238.2	121.1	230.2	113.1
**Arginine**	323.2	121.1	315.2	113.1
**Asparagine**	281.2	121.1	273.2	113.1
**Aspartic acid**	282.1	121.1	274.1	113.1
**Citruline**	324.2	121.1	316.2	113.1
**Cysteine**	537.2	121.1	521.2	113.1
**Glutamate**	296.2	121.1	288.2	113.1
**Glutamine**	295.2	121.1	287.2	113.1
**Glycine**	224.1	121.1	216.1	113.1
**Histidine**	304.2	121.1	296.2	113.1
**Isoleucine**	280.2	121.1	272.2	113.1
**Leucine**	280.2	121.1	272.2	113.1
**Lysine**	443.3	121.1	427.3	113.1
**Methionine**	298.2	121.1	290.2	113.1
**Ornitine**	429.3	121.1	413.3	113.1
**Phenylalanine**	314.2	121.1	306.2	113.1
**Proline**	264.2	121.1	256.2	113.1
**Serine**	254.2	121.1	246.2	113.1
**Taurine**	274.1	121.1	266.1	113.1
**Threonine**	268.2	121.1	260.2	113.1
**Tryptophane**	353.2	121.1	345.2	113.1
**Tyrosine**	330.2	121.1	322.2	113.1
**Valine**	266.2	121.1	258.2	113.1

3. UHPLC-IM-MS analysis

2.1.1.1.1. Sample handling

The sample handling component was a Waters 2777C sample manager (Waters Corp., Milford, MA, USA) equipped with a 25 μL Hamilton syringe, a 2 μL loop used for full loop injections of prepared sample, and a 2-drawer sample chamber thermo-stated at 4°C with a constant flow of dry nitrogen gas to prevent the buildup of condensation.

2.1.1.1.2. Chromatographic conditions

The chromatography was performed on a Waters NanoAcquity UPLC module (Saint Quentin en Yvelines, France) upgraded to work with 1 mm columns and composed with a binary solvent manager and column heater/cooler module. Separation was carried out at 45 °C using a 1.0 x 100 mm, Acquity UPLC HSS T3 column (Waters), with a particle size of 1.8 μm, equipped with a 0.2 μm prefilter. Urine was eluted from the LC column using the following linear gradient (curve number 6): 0–1 min: 99% A; 1–3 min, 99–85% A; 3–6 min, 85-50% A; 6–9 min, 50-0% A; 9–12 min, 100% B, 12–16 min, 99% A for re-equilibration. Solvent A was water and solvent B was acetonitrile, both solvents contained 0.1% formic acid. The duration of column equilibration was adjusted to provide sufficient retention and chromatographic precision of early eluting species in subsequent analyses at the minimal expense of time. Sample analysis order has been randomized to avoid potential for confounding critical variables with analytical run order effects. Peak splitting and column overload was avoided by using small injection volumes (2 μL) for LC-IM-MS analysis.

2.1.1.1.3. Mass spectrometry

The U-HPLC system was coupled to a hybrid quadrupole orthogonal time-of-flight (TOF) mass spectrometer (SYNAPT G2 HDMS, Waters MS Technologies, Manchester, UK). The mass spectrometer was operated in positive electrospray ionization mode. A mass range of *m*/*z* 50−1200 was used in both modes. The sample cone voltage, extraction cone voltage, source temperature, desolvation temperature, desolvation gas flow and cone gas flow were optimized and were as follows respectively: 25V, 5V, 120°C, 500°C, 400 L/h, 50 L/h. Leucine enkephalin was used as the lock mass $[M+H]^+$ at *m*/*z* 556.2771. Sodium formate solution was used for external instrument calibration.

2.1.1.1.4. Ion mobility

Synapt G2 HDMS (Waters MS Technologies, Manchester, UK) was used in our study for Ion Mobility. It is equipped with a traveling wave "Triwave™" geometry in which the ion mobility cell (IMS T-wave) is placed between two traveling wave ion guides (trap T-wave and transfer T-wave). After ionization in the source and transfer through the quadrupole, the ions arrive at the first traveling-wave ion guide that acts as an ion trap, namely "trap TWIG". In this region, the ions are accumulated before being released in packets and accelerated using the trap-bias voltage to the second cell "IMS-TWIG" for mobility separation. In the IMS-TWIG a traveling wave is continuously applied at a given wave height and velocity to enhance separation through the mobility cell, which is filled with a gas. In this study, the IMS drift gas flow (nitrogen) and the wave velocity settings were assessed and optimized. The helium cell gas flow, wave height, Trap Bias and IMS wave delay were set at 180 mL/min, 40 V, 45 V and 450 μs respectively. The TOF analyzer was operated in the *V* resolution mode with an average mass resolution of m/Δm 20,000 (full-width at half-maximum

definition). Data acquisition of an ion mobility experiment consisted of 200 bins. CCS values, obtained in nitrogen, were experimentally determined using singly charged Poly-DL-alanine oligomers as the TWIM calibrant species for ESI+. CCS values were derived according to previously reported procedures [1]. The ion mobility resolution was ~40 Ω/ΔΩ (fwhm). The N_2 CCS values reported were determined at the apex of the ion-mobility peak. Detailed instrument settings are presented in Table S-2.

2.1.1.1.5. Raw data preprocessing

All LC-IM–MS raw data files data processing, peak detection and peak matching across samples using retention time (tR) correction and chromatographic alignment along with drift time and CCS calculation were performed using Progenesis QI (Waters MS Technologies, Manchester, UK) to yield a data matrix containing retention times, accurate masses, CCS and peak intensities. The preprocessing step resulted in an X-matrix where tR, CCS and *m/z* values were concatenated into ''tR_m/z_CCS'' features (in columns) present in each sample (in rows) with corresponding peak areas.

2.1.1.1.6. Quality Control

Aliquoted 10 µL of each urine sample are mixed together to generate a pooled quality control sample (QCs). QCs and solvent blank samples (mobile phase) were injected sequentially in-between the urine samples. In addition, a dilution series of QC samples (6%, 12.5%, 25%, 50% and 100% original concentration) are used to assess the quality of the extracted features. An analysis sequence is presented in Figure. S-1. Indeed, feature extraction algorithms including automatic peak detection, grouping, and integration often yield a data matrix containing analytical system noise such as mobile phase chemical contaminants signals. Depending on the software and the used parameters for feature extraction, such noise can represent a significant portion of the total number of detected features. Therefore, this may mislead further data analysis such as transformation, normalization and data modeling. Simple noise filtering strategies such as the minimum fraction filter may remove infrequently observed signals within the considered distinct sample classes. In this study, we used a filter strategy in which the features intensity must be correlated to the matrix concentration in a series of diluted QC samples in order to be included in further analysis. Beyond its role as a system noise marker, the dilution series filter is very useful to assess the informative quality of the extracted features. It ensures that the observed signal of a given feature and its relative concentration in the sample are positively correlated. This approach is used to identify feature groups that are not correlated to the gradient of concentration generated by the dilutions series and therefore should not be considered as reliable features. Thus, feature groups with correlation coefficient of less than 0.7 were removed from the dataset. Furthermore, datasets are refined by removal of feature groups that do not meet threshold of peak area measurement precision prior to data analysis. This approach uses RSD values derived from repeated measurements of a pooled QC sample. The threshold was set to RSD<25%. Thus enhancing the biological interpretation of metabolomics data. The system stability assessment has been done using the Principal Component Analysis and assessing the clustering of the QC samples. Figures are shown in supporting information (Figures S-2) presenting the PCA score plot derived from the metabolomics analysis of all the urine samples and QCs replicates. In the PCA score plot, each point corresponds to a different sample or QC. The tightness of the clustering reveals the similarity of the samples (QCs), and thus the system's stability. The QCs are tightly clustered indicating a good instrumental stability over the metabolomics analysis.

Table S2. Instrumental settings for UHPLC-IM-MS analysis.

**Chromatography**		Column Temperature	45 (°C)
		Flow rate	80 (μL/min)
		Injection volume	2 μL
**ESI-MS**		Capillary voltage	2 kV
		Sampling cone voltage	25 V
		Extraction cone voltage	5
		Source temperature	120 (°C)
		Desolvation temperature	500 (°C)
		Desolvation gas flow	400 (liters/h)
		Cone gas flow	50
		Optic mode	Resolution
		MS scan rate	0.2 scan/s
		Lock mass solution	Leu–enk (2 μg/ml)
		Lock mass flow rate	6 μL/min
**Triwave DC**	Trap DC	Entrance	3
		Bias	45
		Trap DC	0
		Exit	3
	IMS DC	Entrance	25
		Helium cell DC	35
		Helium exit	−5
		Bias	3
		Exit	0
	Transfer DC	Entrance	4
		Exit	15
**Gas controls**	IMS gas	Nitrogen	80 (ml/min)
	Helium cell	Nitrogen	180 (ml/min)
**Triwave**	Trap	Wave velocity	300 (m/s)
		Wave height	0.5 (V)
	IMS	Wave velocity	857 (m/s)
		Wave height	40 (V)
	Transfer	Wave velocity	300 (m/s)
		Wave height	5 (V)

4. Data analysis and modeling

Support vector regression normalization method was applied using the MetNormalizer R package [2] before any data analysis, to remove the unwanted intra- and inter-batch measurements analytical variations. The effect of this normalization step on the raw data is shown in Figures S-2, S-3 and S-4 (Supporting information). Then normalized data matrix has been log-transformed and pareto-scaled. All data analyzes and modeling were done using SIMCA 14.0 (MKS DAS, Umeå, Sweden). First, hierarchical cluster analysis has been applied to the data set to get an overview of the clustering trends of samples with similar profiles of variable intensity. Furthermore, multivariate data analysis and modeling is performed using Principal Component Analysis (PCA) as an unsupervised method. PCA was first applied to get an overview of the data and identify potential severe outliers which are defined as observations whose scores mapped outside the Hotelling's T2 ellipse (confidence interval = 0.95) in a cross-validated seven-component model. The DmodX was used to detect moderate outliers [3]. Orthogonal Partial Least-Squares-Discriminant Analysis (OPLS-DA) is used as a supervised method. To select the most relevant features, the training group has been repeatedly split into a training set and a test set. A permutation test (999 iterations) was performed to prevent the OPLS-DA over fitting of the model by comparing diagnostic statistic metrics of the generated model with those of randomly generated models. R2X is the cumulative modeled variation in X (X = features), R2Y is the cumulative modeled variation in Y (Y = sample groups), and Q2Y is the cumulative predicted variation in Y, based on the cross-validation. The range of these parameters is between 0 and 1, where 1 indicates a perfect fit. Furthermore, cross-validated analysis of variance (CV-ANOVA) was systematically performed based on the cross-validated model [4]. The Figure S-2 shows a PCA score plot of the raw data compared to normalized, log-transformed and pareto-scaled data which highlights the importance of these data pretreatment steps before data modeling. The X matrix was 100 x 854 variables. The Y matrix was 100 analyses × 3 groups. Characteristics and validation results from all OPLS-DA models are provided in supplemental material.

5. Feature selection and annotation

To select the most discriminant variables for the separation of groups, S-Plot was used. The S-plot combines the covariances and correlations between the **X** matrix and OPLS scores for a given model component. The covariance values give the magnitude of contribution of a variable while the correlation values reflect the effect and reliability of the variable for the model component scores. Variables with both very high correlation and covariance are important for the explanation power of the model. Furthermore, selection of discriminant variables was achieved using the VIP score procedures for each validated OPLS-DA model [5]. Putative annotation of detected features was performed using both accurate mass comparison using freely available metabolite databases HMDB, LipidBlast, KEGG, and Metlin. Furthermore, CCS values were also compared to the MetCCS database [6].

6. Pathway and network analysis

In order to provide a broader understanding of metabolic changes in MPS I, we also explored the biochemical pathways using a network analysis approach using Mummichog (v.1.0.5) which allows pathway enrichment analyses. The idea behind this metabolic network prediction strategy assumes that metabolite concentration alterations are more likely to occur within a metabolic connected network rather than in a random fashion. This Mummichog python package highlights pathways that are significantly impacted in the studied groups. Significantly impacted biochemical pathways are those exhibiting an adjusted p-value <0.05. For this comparison, we focused on features that significantly changed (511 features with q-values = 0.05 and FDR = 5%).

Mummichog annotates metabolites based on accurate mass *m/z* (5 ppm mass error was used) and tests significant pathway enrichment within a reference metabolic network using a Fisher's exact test [7]. The matched candidates were then mapped to reference human metabolic networks from the KEGG, MetaCyc, Recon and Edinburgh Human Metabolic Network. The null distribution in pathway analysis was obtained from 1000 set of randomly permutated *m/z* lists draw from all features detected in the whole metabolomic dataset and modeled by Gamma distribution. To protect against incorrect pathway selection, redundant pathways or those enriched by less than two metabolites were excluded. MetaboAnalyst [8] has been used for Metabolite Set Enrichment Analysis using the amino acid concentration matrix.

Table S3. The normalized concentrations of free amino acids in urine samples of the five studied groups. (µM / mM creatinine)

	Control		MPS IIIA		MPS IIIB		MPS IIIC		MPS IIID	
	**Mean**	**Range**	**Mean**	**Range**	**Mean**	**Range**	**Mean**	**Range**	**Mean**	**Range**
**L-Alanine**	22.4	0.1 - 83.3	43.45	10.5 - 117.6	45.01	23.1 - 91.2	31.78	5.9 - 160.5	40.12	22.8 - 81.3
**L-Arginine**	2.2	0 - 27.9	13.59	1.1 - 35.9	17.67	7.6 - 42.8	10.27	2.2 - 21	11.53	5 - 35.4
**L-Asparagine**	11.9	0 - 89.6	10.52	0.9 - 44.6	16.11	1.4 - 35	8.48	0.9 - 154.6	10.39	2.1 - 46.2
**L-Aspartic acid**	1.2	0 - 8.8	7.50	0.5 - 47.1	8.77	0.6 - 30.7	2.35	0.4 - 157	0.85	0.1 - 7.5
**L-Citrulline**	1.9	0 - 17.1	0.71	0 - 4.6	0.96	0.1 - 4.9	0.27	0 - 4.7	0.18	0 - 1.2
**L-Cystathionine**	2.9	0 - 9.8	1.04	0 - 5.9	0.91	0.1 - 5.2	1.74	0.6 - 16	2.79	1.3 - 5.6
**L-Cystine**	4.8	0 - 22.7	3.94	0.2 - 13.8	6.01	0.6 - 15.1	3.51	0.1 - 16.3	5.35	2.6 - 15.9
**L-Glutamic acid**	3.5	0 - 19.5	4.05	1.5 - 59.6	2.58	0.9 - 8.6	1.64	0.1 - 10	1.71	0.1 - 9.6
**L-Glutamine**	38.4	0.2 - 138.8	48.62	2.4 - 189.3	84.69	1.8 - 167.1	34.75	0.1 - 341.2	47.32	3.2 - 491.8
**L-Glycine**	112.3	0.5 - 448.5	175.75	45.5 - 735.5	167.04	41.8 - 355.7	119.60	22.1 - 469.8	216.42	60.9 - 472.3
**L-Histidine**	52.2	0.3 - 147.7	99.82	29.4 - 351.8	106.86	30.2 - 247.9	70.99	13.2 - 458.8	81.81	14.9 - 391.3
**L-isoleucine**	1.8	0 - 11.8	0.96	0 - 4.1	1.69	0.2 - 3.5	0.69	0.1 - 3.7	1.10	0.2 - 4.9
**L-Leucine**	3.3	0 - 19.2	2.96	0.2 - 11.4	4.42	0.5 - 9.4	1.76	0.4 - 11.2	2.71	1.1 - 11.5
**L-Lysine**	13.6	0.2 - 70	14.31	2.6 - 140.7	14.88	2.5 - 105	8.23	1.1 - 170.5	15.96	4 - 34.9
**L-Methionine**	0.8	0 - 6.1	0.29	0 - 1.6	0.36	0.1 - 1.7	0.11	0 - 2.1	0.22	0 - 7.7
**L-Ornithine**	5.1	0 - 91	1.01	0 - 6	0.62	0.1 - 4.1	0.29	0 - 3.7	0.25	0 - 4.1
**L-Phenylalanine**	5.1	0 - 23.7	7.14	2.4 - 24.1	6.70	0.2 - 14.3	4.41	0.4 - 27	3.76	0.2 - 31.5
**L-Proline**	2	0 - 14.1	2.04	0.7 - 10	2.34	0.9 - 6.9	1.13	0.4 - 3.3	1.43	1 - 3.6
**L-Serine**	32.2	0 - 119.8	20.37	0 - 120.7	46.24	1.5 - 93.1	13.05	0.1 - 252.6	19.28	0.2 - 143.8
**L-Taurine**	33.3	0 - 106.8	56.50	13.3 - 285.5	31.54	3.7 - 140	32.70	8.9 - 137.9	24.42	2.5 - 114
**L-Threonine**	13.2	0.8 - 71	16.28	1.5 - 50.7	20.50	1.1 - 52.8	7.93	0.1 - 157	9.07	0.2 - 69.7
**L-Tryptophane**	4.4	0 - 9.6	6.89	3.3 - 20.4	6.25	0.1 - 16.9	3.63	0.1 - 28.6	3.33	0.2 - 18.7
**L-Tyrosine**	8.3	0 - 39.4	9.81	3.6 - 39.6	12.73	1.2 - 35	5.73	0 - 43.7	5.86	0.4 - 54.4
**L-Valine**	4.2	0 - 22.6	3.49	0.2 - 15.9	4.50	0.2 - 10.9	2.29	0.4 - 10.5	3.45	1.1 - 17.9

Table S4. Data for venn diagram of the significant pathways retrieved from metabolomics approaches and *in silico* systems biology approach from Salazar DA et al [9].

Data	N°	Pathways
MPS III *vs in silico vs* MPS IIIA *vs* MPS IIIB *vs* MPS IIIC *vs* MPS IIID	2	UREA CYCLE<br>ARGININE AND PROLINE METABOLISM
MPS IIIA *vs* MPS IIIB *vs* MPS IIIC	3	ASPARTATE METABOLISM<br>MALATE-ASPARTATE SHUTTLE<br>BETA-ALANINE METABOLISM
MPS IIIA *vs* MPS IIIB	4	AMMONIA RECYCLING<br>SELENOAMINO ACID METABOLISM<br>GLUCOSE-ALANINE CYCLE<br>ALANINE METABOLISM
MPS IIIB *vs* MPS IIID	1	PORPHYRIN METABOLISM
MPS III *vs in silico vs* MPS IIIB	2	GLUTAMATE METABOLISM<br>TYROSINE METABOLISM
MPS IIIB	4	CATECHOLAMINE BIOSYNTHESIS<br>PURINE METABOLISM<br>GLYCINE, SERINE AND THREONINE METABOLISM<br>PYRIMIDINE METABOLISM
MPS IIIC	1	BETAINE METABOLISM
MPS III *vs in silico*	76	TRANSPORT, PEROXISOMAL KERATAN SULFATE DEGRADATION<br>VITAMIN D METABOLISM<br>OXIDATIVE PHOSPHORYLATION<br>STARCH AND SUCROSE METABOLISM<br>PURINE CATABOLISM<br>PYRIMIDINE CATABOLISM<br>AMINOSUGAR METABOLISM<br>GLUTATHIONE METABOLISM<br>EICOSANOID METABOLISM<br>TRANSPORT, ENDOPLASMIC RETICULAR<br>TRANSPORT, GOLGI APPARATUS<br>BIOTIN METABOLISM<br>TRANSPORT, EXTRACELLULAR<br>VITAMIN C METABOLISM<br>PYRIMIDINE SYNTHESIS<br>CITRIC ACID CYCLE<br>COA SYNTHESIS<br>TRANSPORT, MITOCHONDRIAL<br>PYRUVATE METABOLISM<br>PENTOSE PHOSPHATE PATHWAY<br>NAD METABOLISM<br>TRIACYLGLYCEROL SYNTHESIS<br>ROS DETOXIFICATION<br>LYSINE METABOLISM<br>D-ALANINE METABOLISM<br>GLYCINE, SERINE, ALANINE AND THREONINE METABOLISM<br>GLYCOSPHINGOLIPID METABOLISM<br>TAURINE AND HYPOTAURINE METABOLISM<br>VITAMIN A METABOLISM<br>GLYOXYLATE AND DICARBOXYLATE METABOLISM<br>STEROID METABOLISM<br>CHOLESTEROL METABOLISM<br>ALANINE AND ASPARTATE METABOLISM<br>CYSTEINE METABOLISM<br>THIAMINE METABOLISM<br>NUCLEOTIDE INTERCONVERSION<br>ANDROGEN AND ESTROGEN SYNTHESIS AND METABOLISM<br>FRUCTOSE AND MANNOSE METABOLISM<br>HEME SYNTHESIS<br>DIETARY FIBER BINDING<br>TETRAHYDROBIOPTERIN METABOLISM<br>O-GLYCAN SYNTHESIS<br>COA CATABOLISM<br>VALINE, LEUCINE, AND ISOLEUCINE METABOLISM<br>BUTANOATE METABOLISM<br>SPHINGOLIPID METABOLISM<br>TRYPTOPHAN METABOLISM<br>HISTIDINE METABOLISM<br>VITAMIN B6 METABOLISM<br>GLYCEROPHOSPHOLIPID METABOLISM<br>FOLATE METABOLISM<br>FATTY ACID SYNTHESIS<br>VITAMIN B2 METABOLISM<br>SQUALENE AND CHOLESTEROL SYNTHESIS<br>UNASSIGNED<br>TRANSPORT, NUCLEAR<br>METHIONINE AND CYSTEINE METABOLISM<br>PHOSPHATIDYLINOSITOL PHOSPHATE METABOLISM<br>C5-BRANCHED DIBASIC ACID METABOLISM<br>GLYCOLYSIS/GLUCONEOGENESIS<br>BLOOD GROUP SYNTHESIS<br>TRANSPORT, LYSOSOMAL<br>PHENYLALANINE METABOLISM<br>FATTY ACID OXIDATION<br>PROPANOATE METABOLISM<br>N-GLYCAN DEGRADATION<br>INOSITOL PHOSPHATE METABOLISM<br>PURINE SYNTHESIS<br>N-GLYCAN SYNTHESIS<br>R GROUP SYNTHESIS<br>KERATAN SULFATE SYNTHESIS<br>BILE ACID SYNTHESIS<br>GALACTOSE METABOLISM<br>MISCELLANEOUS<br>EXCHANGE/DEMAND REACTION

Figure S1. Illustration of the analysis sequence order. Ten µL of each urine sample are mixed together to generate a pooled quality control sample (QCs). QCs and solvent blank samples (mobile phase) were injected sequentially in-between the urine samples. In addition, a dilution series of QC samples (6%, 12.5%, 25%, 50% and 100% of the original concentration) are used to assess the quality of the extracted features. A conditioning step is used to condition the column using ten QC injections. Sample injection order has been orthogonalized.

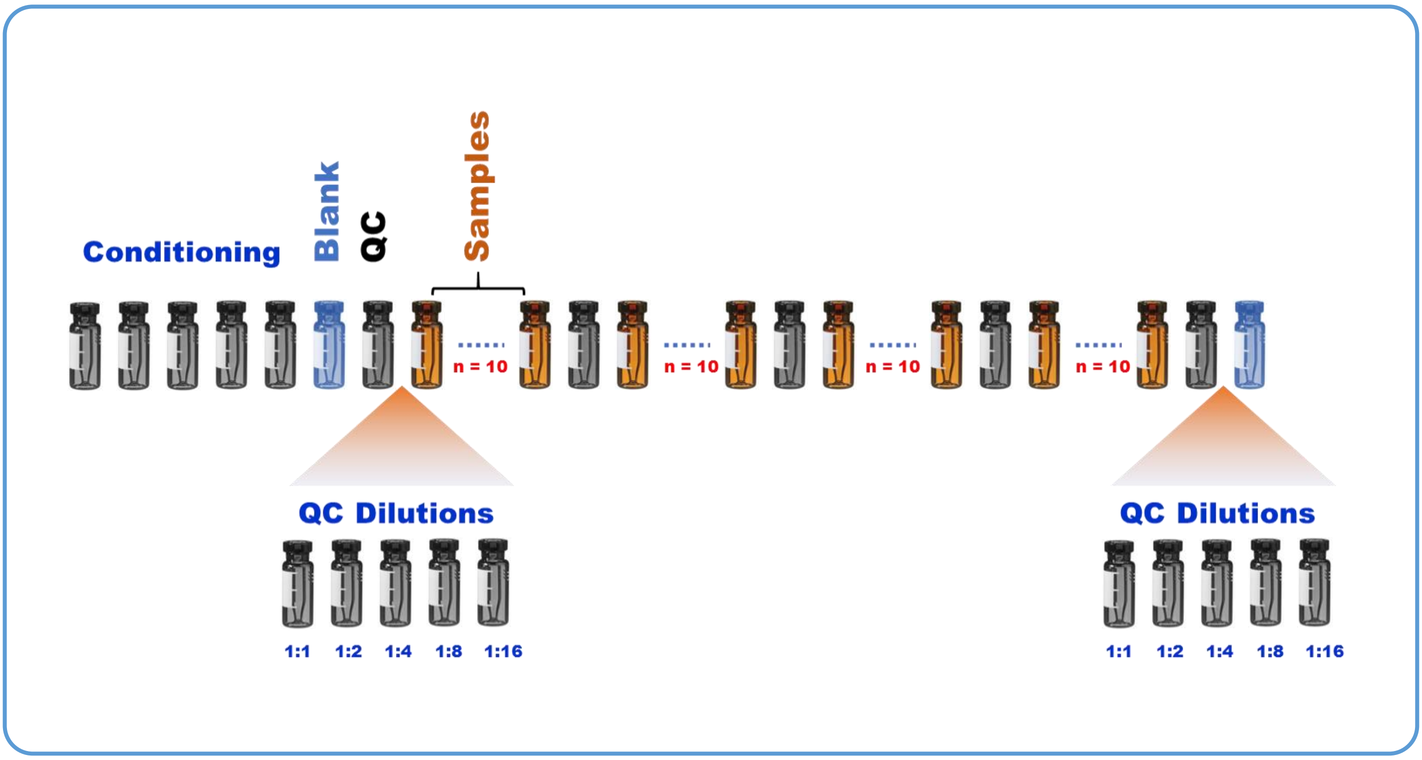

Figure S2. OPLSDA model validation including the three groups: MPS IIIA, MPS IIIB, MPS IIIC, MPS IIID and Controls.
Below the figure, model parameters and CV-ANOVA results are presented.

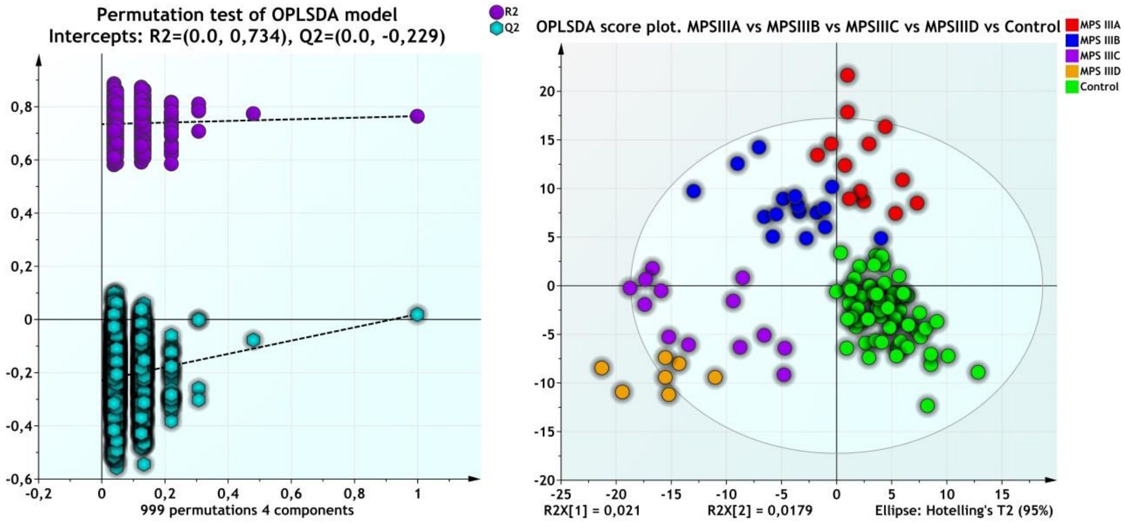

MPS IIIA vs MPS IIIB vs MPS IIIC vs MPS IIID vs Controls - OPLSDA model parameters.

Component	R2X	R2X(cum)	Eigenvalue	R2	R2(cum)	Q2	Limit	Q2(cum)	R2Y	R2Y(cum)
Model		0.333			0.772			0.134		1
Predictive		0.0639			0.772			0.134		1
P1	0.021	0.021	2.46	0.236	0.236	0.0991	0.01	0.0991	0.304	0.304
P2	0.0179	0.0389	2.09	0.205	0.442	0.0542	0.01	0.153	0.248	0.552
P3	0.0145	0.0534	1.7	0.159	0.601	0.00279	0.01	0.156	0.219	0.771
P4	0.0105	0.0639	1.23	0.172	0.772	-0.022	0.01	0.134	0.229	1
Orthogonal in X(OPLS)		0.27			0					
O1	0.209	0.209	24.5	0	0					
O2	0.0396	0.249	4.63	0	0					
O3	0.0206	0.27	2.41	0	0					

MPS IIIA vs MPS IIIB vs MPS IIIC vs MPS IIID vs Controls - CV-ANOVA test results.

	SS	DF	MS	F	*p*	SD
Total corr.	327	327	1			1
Regression	29.7924	18	1.65513	1.7208	0.0348787	1.28652
Residual	297.208	309	0.961837			0.980733

Figure S3. OPLSDA model validation for MPS IIIA *vs*. Control.
Below the figure, model parameters and CV-ANOVA results are presented.

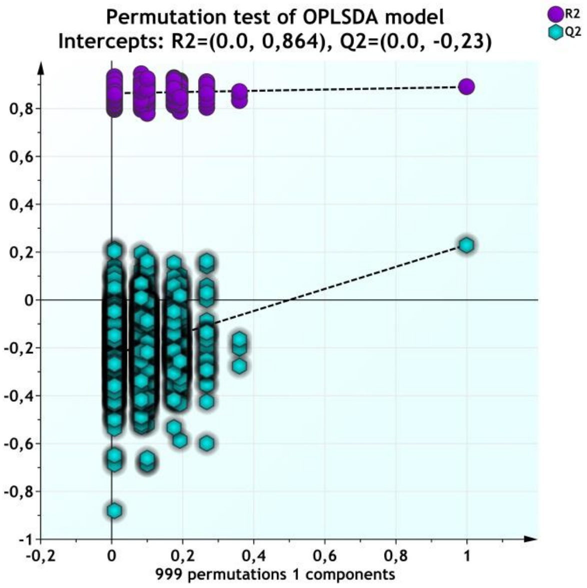

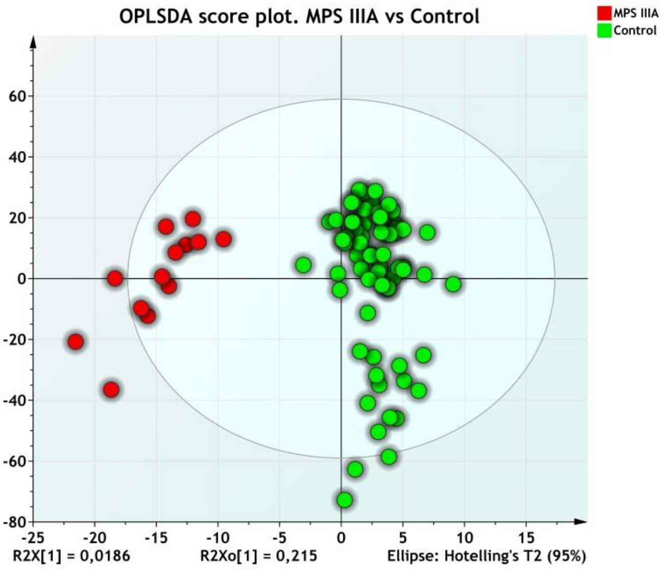

MPS IIIA *vs*. Control - OPLSDA model parameters.

Component	R2X	R2X(cum)	Eigenvalue	R2	R2(cum)	Q2	Limit	Q2(cum)	R2Y	R2Y(cum)
Model		0.267			0.89			0.231		1
Predictive		0.0186			0.89			0.231		1
P1	0.0186	0.0186	1.5	0.89	0.89	0.231	0.01	0.231	1	1
Orthogonal in X(OPLS)		0.248			0					
O1	0.215	0.215	17.4	0	0					
O2	0.033	0.248	2.67	0	0					

MPS IIIA *vs*. Control - CV-ANOVA test results.

	SS	DF	MS	F	*p*	SD
Total corr.	80	80	1			1
Regression	18.4697	6	3.07828	3.70213	0.00284566	1.7545
Residual	61.5303	74	0.83149			0.911861

Figure S4. OPLSDA model validation for MPS IIIB *vs*. Control

Below the figure, model parameters and CV-ANOVA results are presented.

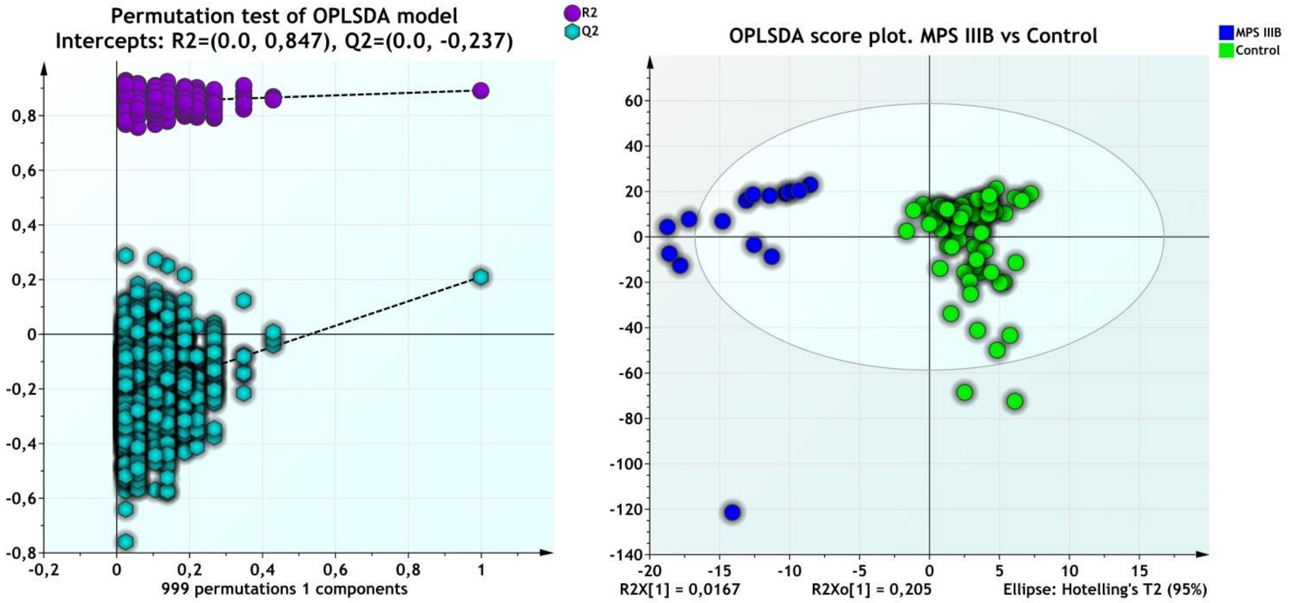

MPS IIIB *vs*. MPSIT - OPLSDA model parameters.

Component	R2X	R2X(cum)	Eigenvalue	R2	R2(cum)	Q2	Limit	Q2(cum)	R2Y	R2Y(cum)
Model		0.288			0.892			0.21		1
Predictive		0.0167			0.892			0.21		1
P1	0.0167	0.0167	1.38	0.892	0.892	0.21	0.01	0.21	1	1
Orthogonal in X(OPLS)		0.271			0					
O1	0.205	0.205	17	0	0					
O2	0.0666	0.271	5.53	0	0					

MPS IIIB *vs*. MPSIT - CV-ANOVA test results.

	SS	DF	MS	F	*p*	SD
Total corr.	82	82	1			1
Regression	17.2511	6	2.87519	3.3748	0.00528414	1.69564
Residual	64.7489	76	0.851959			0.923016

Figure S5. OPLSDA model validation for MPS IIIC *vs*. Control

Below the figure, model parameters and CV-ANOVA results are presented.

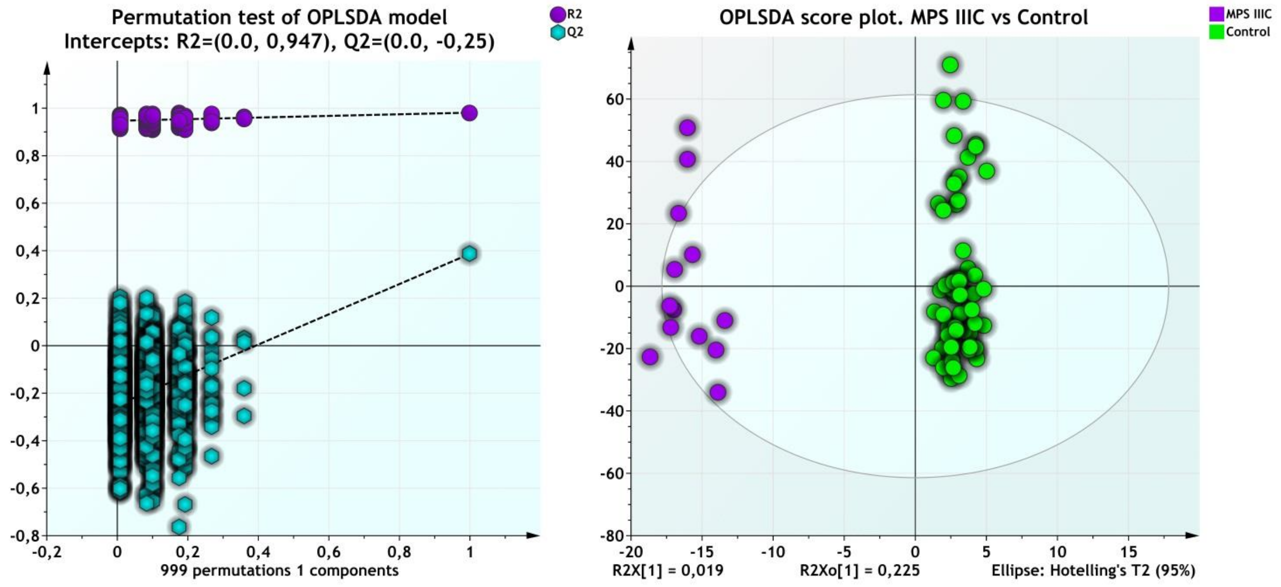

MPS IIIC *vs*. Control - OPLSDA model parameters.

Component	R2X	R2X(cum)	Eigenvalue	R2	R2(cum)	Q2	Limit	Q2(cum)	R2Y	R2Y(cum)
Model		0.3			0.981			0.387		1
Predictive		0.019			0.981			0.387		1
P1	0.019	0.019	1.54	0.981	0.981	0.387	0.01	0.387	1	1
Orthogonal in X(OPLS)		0.281			0					
O1	0.225	0.225	18.3	0	0					
O2	0.0337	0.259	2.73	0	0					
Q3	0.0222	0.281	1.8	0	0					

MPS IIIC *vs*. Control - CV-ANOVA test results.

	SS	DF	MS	F	*p*	SD
Total corr.	80	80	1			1
Regression	30.9465	8	3.86831	5.67784	1.35874e-005	1.9668
Residual	49.0535	72	0.681299			0.825408

Figure S6. OPLSDA model validation for MPS IIID *vs*. Control

Below the figure, model parameters and CV-ANOVA results are presented.

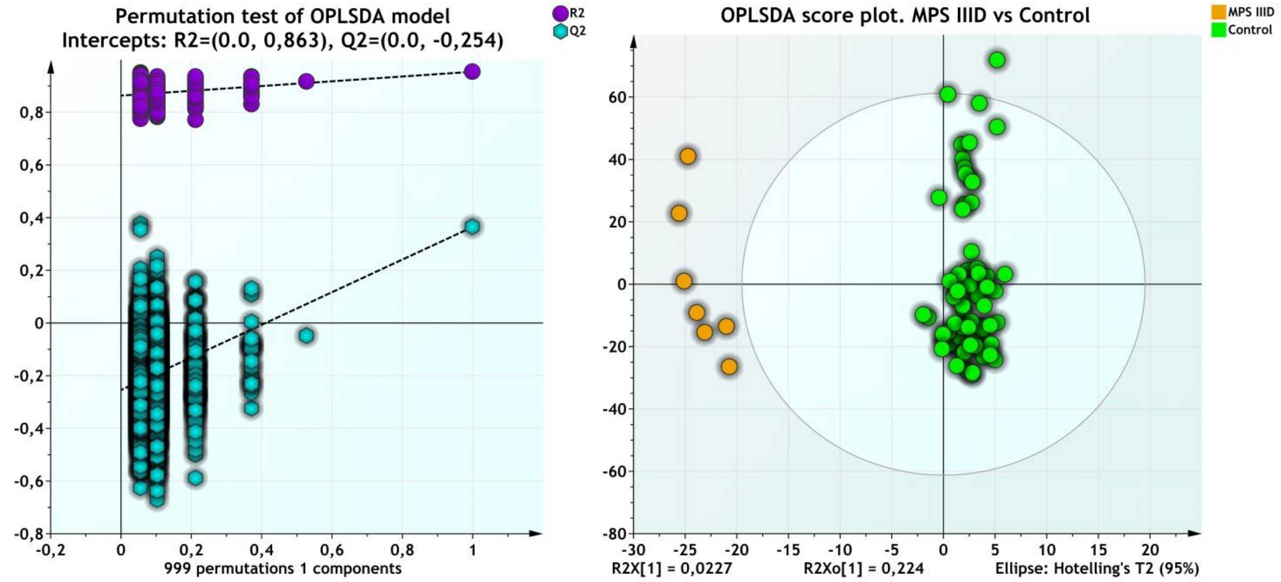

MPS IIID *vs*. Control - OPLSDA model parameters.

Component	R2X	R2X(cum)	Eigenvalue	R2	R2(cum)	Q2	Limit	Q2(cum)	R2Y	R2Y(cum)
Model		0.274			0.954			0.365		1
Predictive		0.0227			0.954			0.365		1
P1	0.0227	0.0227	1.71	0.954	0.954	0.365	0.01	0.365	1	1
Orthogonal in X(OPLS)		0.251			0					
O1	0.224	0.224	16.8	0	0					
O2	0.0275	0.251	2.06	0	0					

MPS IIID *vs*. Control - CV-ANOVA test results.

	SS	DF	MS	F	*p*	SD
**Total corr.**	74	74	1			1
**Regression**	26.9998	6	4.49997	6.51058	1.83729e-005	2.12131
**Residual**	47.0002	68	0.691179			0.831372

Figure S7. Boxplots of selected discriminant features in the assessed groups: MPS IIIA, MPS IIIB, MPS IIIC, MPS IIID and Control samples.

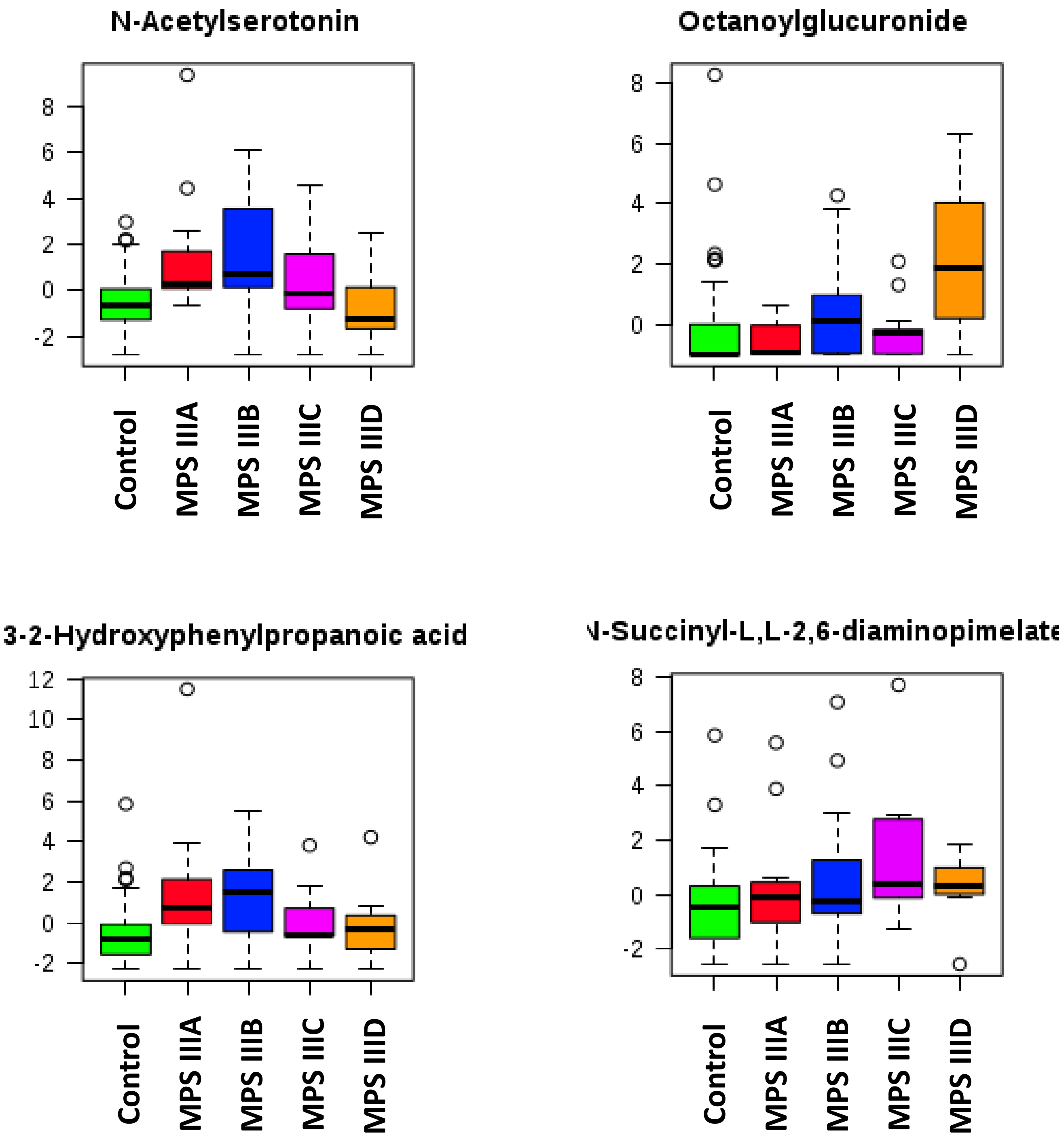

Figure S8. Boxplots of amino acid concentrations across the five studied groups: MPS IIIA, MPS IIIB, MPS IIIC, MPS IIID and Control samples. (*: *p*<0.05, **: *p*<0.01, ***: *p*<0.001)

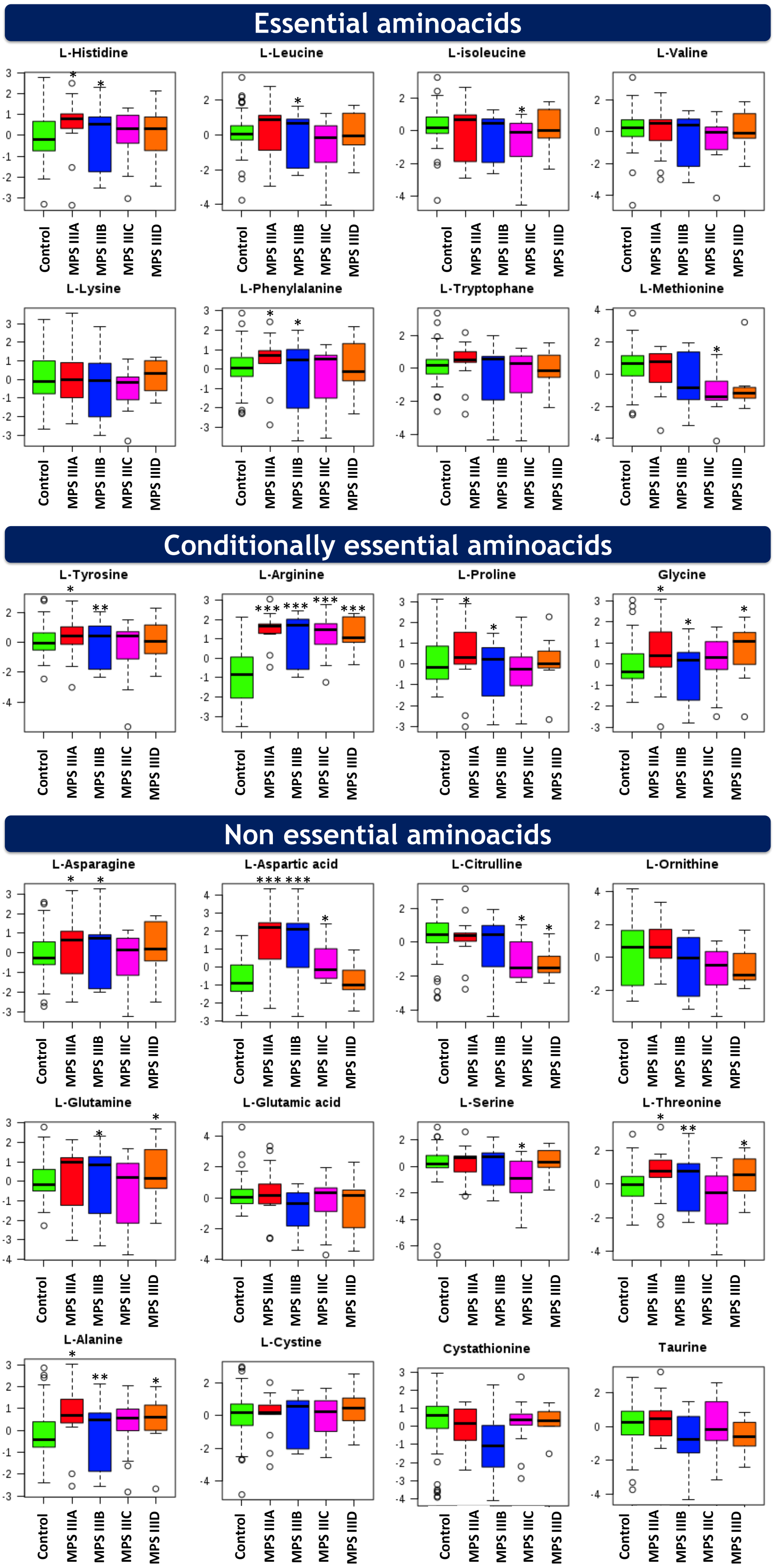

Figure S9. Area under the receiver operating characteristic (ROC) curves, comparing diagnostic performance of the most significant quantified amino acid Arginine to differentiate the different MPS III subtypes Control samples. A comparison of different combinations of the different amino acids using a PLSDA model with three components is also shown. Combining amino acids does not show significant improvement in AUC.
AUC, Area under the curve. False positive rate = 100 – Specificity. True positive rate = Sensitivity.

A. MPS IIIA *vs* Control

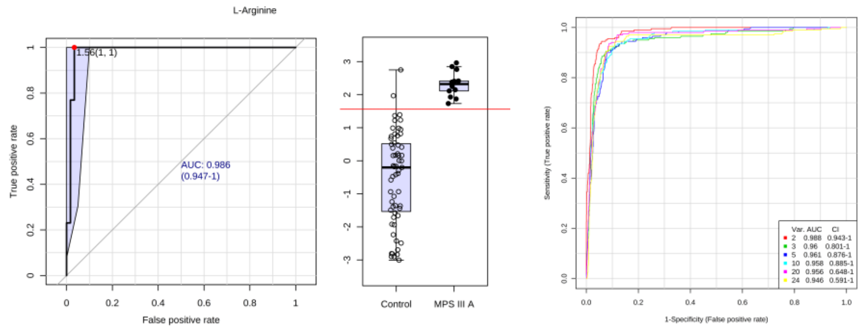

B. MPS IIIB *vs* Control

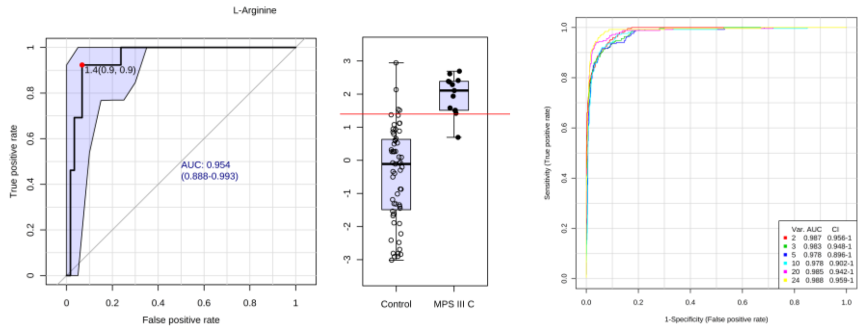

C. MPS IIIC *vs* Control

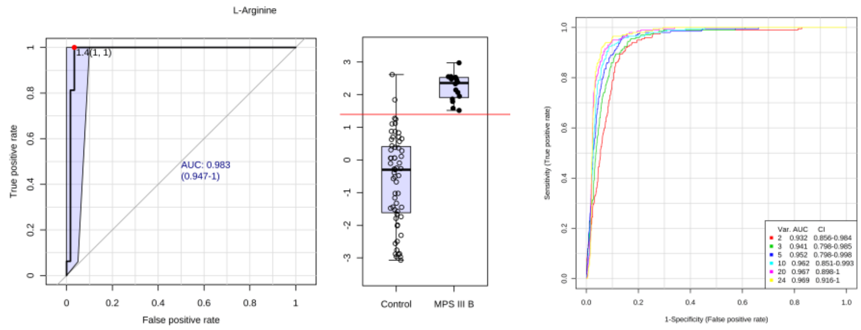

D. MPS IIID *vs* Control

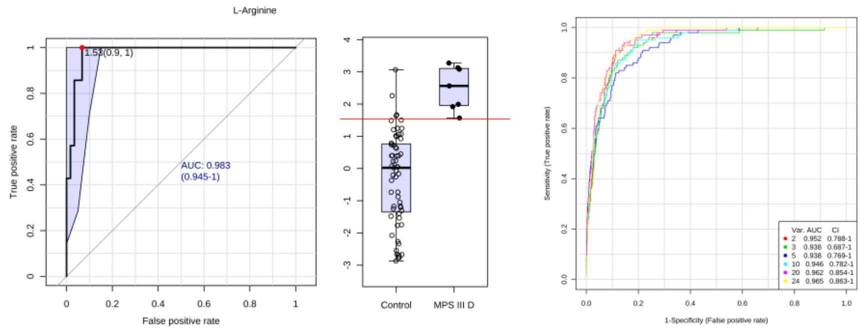

Figure S10. Pathways of arginine metabolism and its connections to urea cycle. Enzymes that catalyze the indicated reactions are as follows: 1: argininosuccinic lyase, 2: NO synthases. 3: arginine:glycine amidinotransferase. 4: arginase. 5, arginine decarboxylase. 6: agmatinase (agmatine ureohydrolase). 7: guanidinoacetate N-methyltransferase. 9: ornithine aminotransferase. 10: pyrroline- 5-carboxylate reductase. 11: pyrroline- 5-carboxylate dehydrogenase. 12: glutamate dehydrogenase. 13: alanine aminotransferase, aspartate aminotransferase, or branched-chain amino acid aminotransferase. 14: glutamine synthetase. 15: glutaminase. 16: ornithine decarboxylase. 17: spermidine synthase. 18: spermine synthase. 19: diamine oxidase. 20, aldehyde dehydrogenase. 21: glutamate decarboxylase. Complete oxidation of arginine-derived a-ketoglutarate occurs via the citric acid cycle. Step 8 is a spontaneous, nonenzymatic reaction. DCAM: decarboxylated S-adenosylmethionine. Glu: L-glutamate. MTA: methylthioadenosine. SAHC: S-adenosylhomocysteine. SAM: S-adenosylmethionine. aKG: a-ketoglutarate. The metabolites that were measured in the present study are shown in red boxes. Elevated metabolites in MPSI urine are highlighted in orange. For a more convenient highlight of the metabolites, each MPS III subtype is depicted in a single figure hereafter.

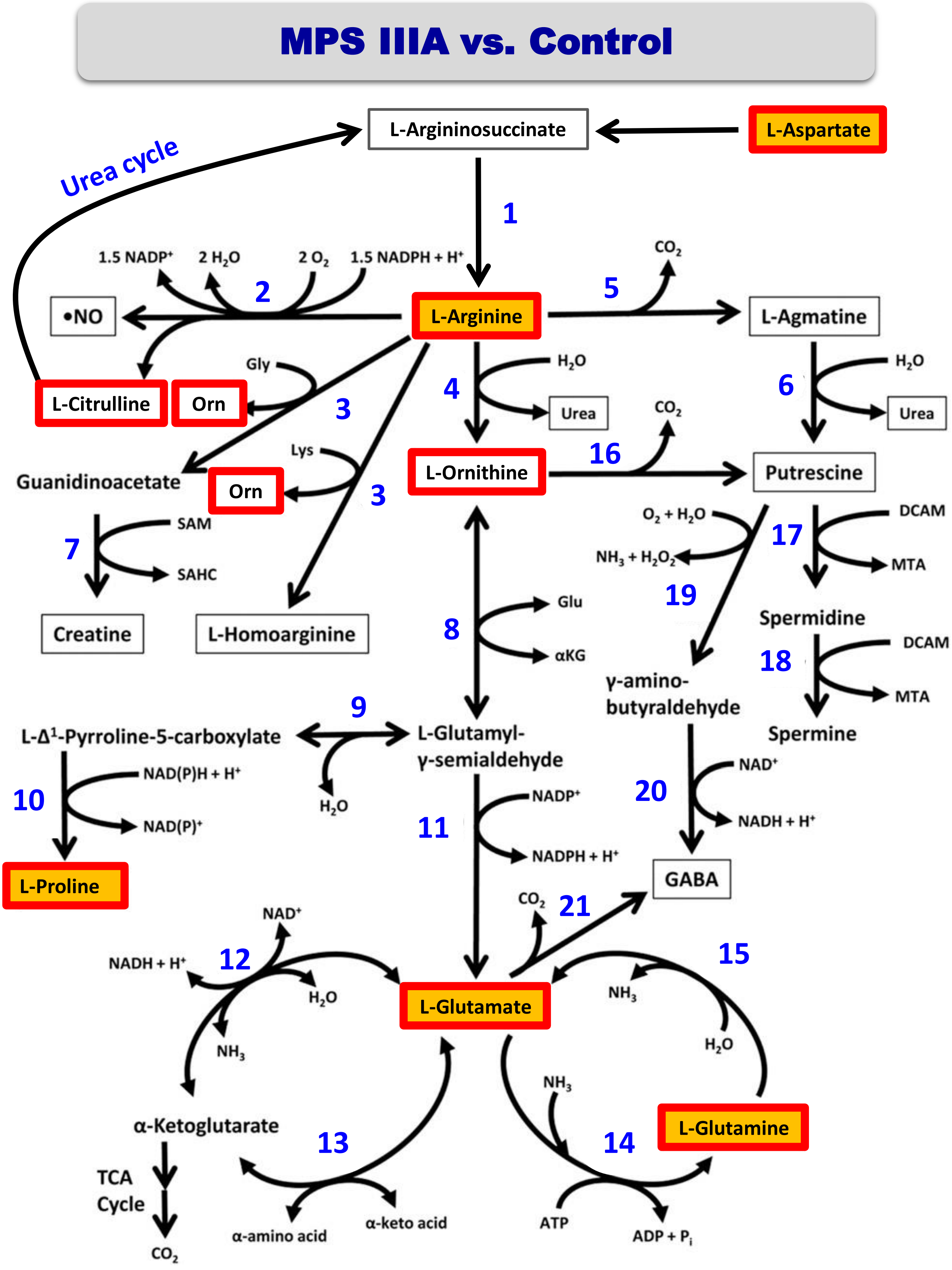
MPS IIIA vs. Control
L-Argininosuccinate
L-Aspartate
Urea cycle
1
1.5 NADP+
2 H2O
2 O2
1.5 NADPH + H+
2
•NO
L-Arginine
5
CO2
L-Agmatine
L-Citrulline
Orn
Gly
3
4
H2O
Urea
6
H2O
Urea
Lys
Guanidinoacetate
Orn
3
L-Ornithine
16
CO2
Putrescine
7
SAM
SAHC
O2 + H2O
NH3 + H2O2
17
DCAM
MTA
19
Creatine
L-Homoarginine
8
Glu
αKG
Spermidine
18
DCAM
MTA
γ-amino-
butyraldehyde
9
L-Δ1-Pyrroline-5-carboxylate
L-Glutamyl-
γ-semialdehyde
Spermine
10
NAD(P)H + H+
NAD(P)+
H2O
NADP+
11
NADPH + H+
20
NAD+
NADH + H+
L-Proline
GABA
CO2
21
NAD+
NADH + H+
12
H2O
NH3
L-Glutamate
15
NH3
H2O
NH3
α-Ketoglutarate
13
14
L-Glutamine
TCA
Cycle
CO2
α-amino acid
α-keto acid
ATP
ADP + Pi

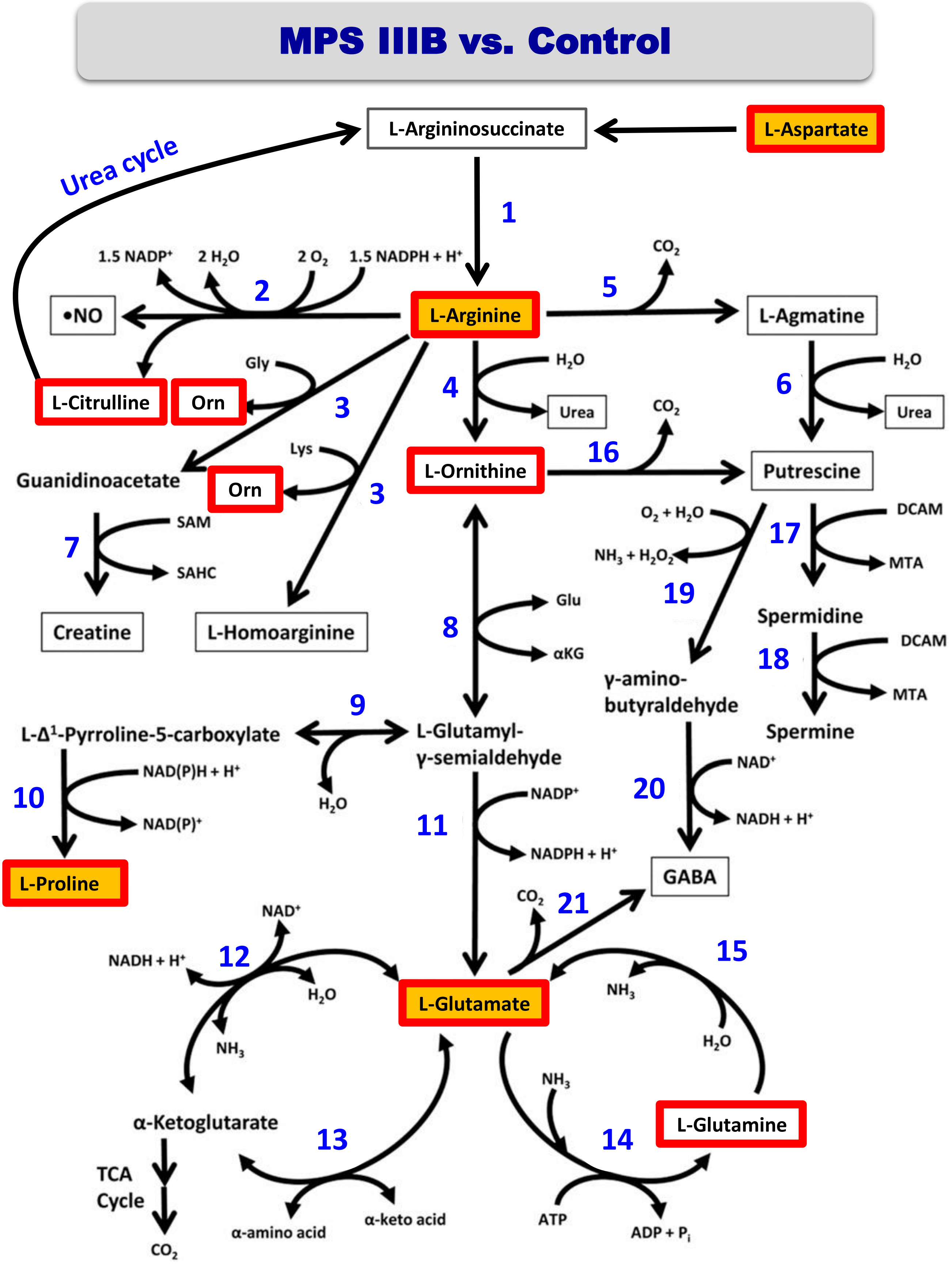
MPS IIIB vs. Control
L-Argininosuccinate
L-Aspartate
Urea cycle
1
1.5 NADP⁺
2 H₂O
2 O₂
1.5 NADPH + H⁺
2
•NO
L-Arginine
5
CO₂
L-Agmatine
L-Citrulline
Orn
Gly
3
4
H₂O
Urea
6
H₂O
Urea
Lys
Guanidinoacetate
Orn
3
L-Ornithine
16
CO₂
Putrescine
7
SAM
SAHC
O₂ + H₂O
NH₃ + H₂O₂
17
DCAM
MTA
19
Creatine
L-Homoarginine
8
Glu
αKG
Spermidine
18
DCAM
MTA
γ-amino-butyraldehyde
9
L-Δ¹-Pyrroline-5-carboxylate
L-Glutamyl-γ-semialdehyde
Spermine
10
NAD(P)H + H⁺
NAD(P)⁺
H₂O
11
NADP⁺
NADPH + H⁺
20
NAD⁺
NADH + H⁺
L-Proline
GABA
CO₂
21
NAD⁺
NADH + H⁺
12
H₂O
NH₃
L-Glutamate
15
NH₃
H₂O
NH₃
α-Ketoglutarate
13
TCA Cycle
CO₂
α-amino acid
α-keto acid
14
L-Glutamine
ATP
ADP + Pᵢ

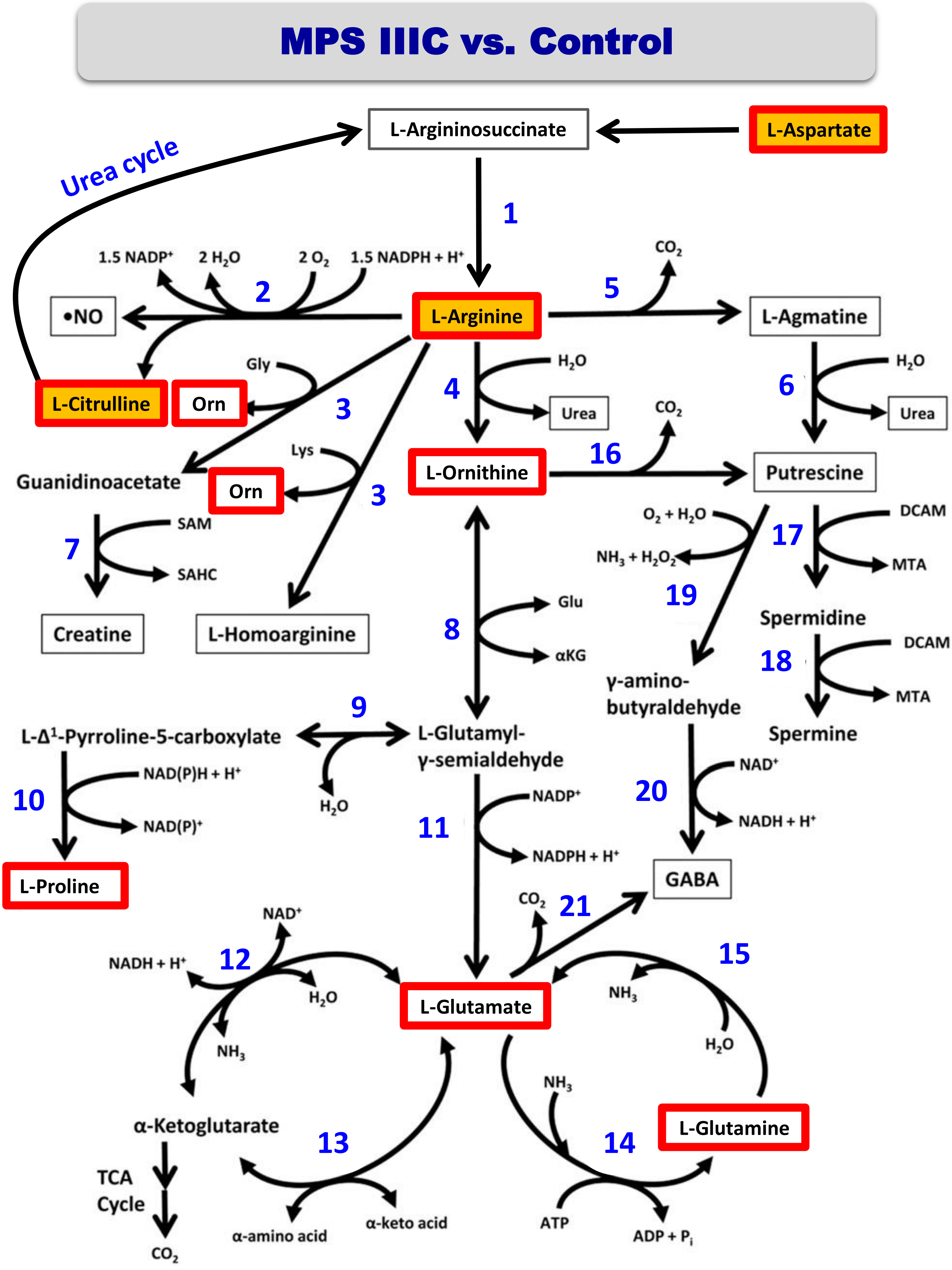
MPS IIIC vs. Control
L-Argininosuccinate
L-Aspartate
Urea cycle
1
1.5 NADP+
2 H2O
2 O2
1.5 NADPH + H+
2
CO2
5
•NO
L-Arginine
L-Agmatine
Gly
H2O
H2O
L-Citrulline
Orn
3
4
Urea
6
Urea
Lys
CO2
16
Guanidinoacetate
Orn
3
L-Ornithine
Putrescine
SAM
O2 + H2O
DCAM
7
NH3 + H2O2
17
SAHC
MTA
19
Glu
Creatine
L-Homoarginine
8
Spermidine
αKG
DCAM
18
γ-amino-
butyraldehyde
MTA
9
L-Δ1-Pyrroline-5-carboxylate
L-Glutamyl-
γ-semialdehyde
Spermine
NAD(P)H + H+
NAD+
10
H2O
NADP+
20
NAD(P)+
11
NADH + H+
NADPH + H+
L-Proline
GABA
CO2
21
NAD+
15
NADH + H+
12
H2O
L-Glutamate
NH3
NH3
H2O
NH3
α-Ketoglutarate
13
14
L-Glutamine
TCA
Cycle
α-keto acid
ATP
α-amino acid
ADP + Pi
CO2

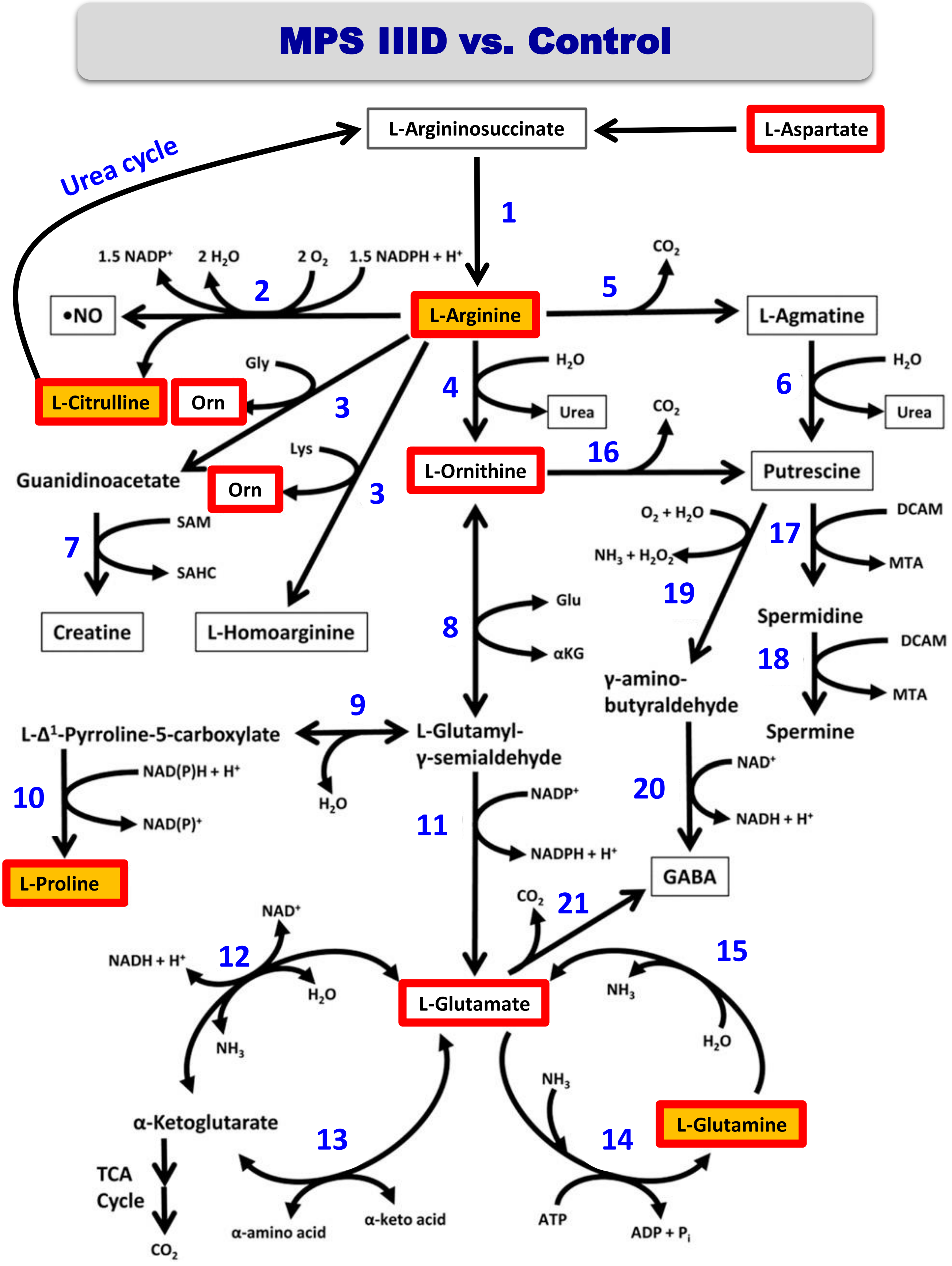
MPS IIID vs. Control
L-Argininosuccinate
L-Aspartate
Urea cycle
1
1.5 NADP⁺
2 H₂O
2 O₂
1.5 NADPH + H⁺
2
•NO
L-Arginine
5
CO₂
L-Agmatine
L-Citrulline
Orn
Gly
3
4
H₂O
Urea
6
H₂O
Urea
Guanidinoacetate
Lys
Orn
3
L-Ornithine
16
CO₂
Putrescine
7
SAM
SAHC
O₂ + H₂O
NH₃ + H₂O₂
17
DCAM
MTA
Creatine
L-Homoarginine
8
Glu
αKG
19
Spermidine
18
DCAM
MTA
γ-amino-butyraldehyde
Spermine
9
L-Δ¹-Pyrroline-5-carboxylate
L-Glutamyl-γ-semialdehyde
10
NAD(P)H + H⁺
NAD(P)⁺
H₂O
11
NADP⁺
NADPH + H⁺
20
NAD⁺
NADH + H⁺
L-Proline
GABA
CO₂
21
NAD⁺
NADH + H⁺
12
H₂O
NH₃
L-Glutamate
15
NH₃
H₂O
NH₃
α-Ketoglutarate
13
14
L-Glutamine
TCA Cycle
CO₂
α-amino acid
α-keto acid
ATP
ADP + Pᵢ

References

1. Paglia G, Angel P, Williams JP, et al. Ion mobility-derived collision cross section as an additional measure for lipid fingerprinting and identification. *Analytical chemistry* 2015; **87**(2): 1137-44.
2. Shen X, Gong X, Cai Y, et al. Normalization and integration of large-scale metabolomics data using support vector regression. *Metabolomics* 2016; **12**(5): 89.
3. Eriksson L, Trygg J, Wold S. A chemometrics toolbox based on projections and latent variables. *Journal of Chemometrics* 2014; **28**(5): 332-46.
4. Eriksson L, Trygg J, Wold S. CV-ANOVA for significance testing of PLS and OPLS® models. *Journal of Chemometrics* 2008; **22**(11-12): 594-600.
5. Galindo-Prieto B, Eriksson L, Trygg J. Variable influence on projection (VIP) for orthogonal projections to latent structures (OPLS). *Journal of Chemometrics* 2014.
6. Zhou Z, Xiong X, Zhu ZJ. MetCCS Predictor: a web server for predicting collision cross-section values of metabolites in ion mobility-mass spectrometry based metabolomics. *Bioinformatics (Oxford, England)* 2017.
7. Li S, Park Y, Duraisingham S, et al. Predicting network activity from high throughput metabolomics. *PLoS Comput Biol* 2013; **9**: e1003123.
8. Xia J, Sinelnikov IV, Han B, Wishart DS. MetaboAnalyst 3.0-making metabolomics more meaningful. *Nucleic acids research* 2015.
9. Salazar DA, Rodriguez-Lopez A, Herreno A, et al. Systems biology study of mucopolysaccharidosis using a human metabolic reconstruction network. *Molecular genetics and metabolism* 2016; **117**(2): 129-39.
